# The noisy basis of morphogenesis: Mechanisms and mechanics of cell sheet folding inferred from developmental variability

**DOI:** 10.1371/journal.pbio.2005536

**Published:** 2018-07-12

**Authors:** Pierre A. Haas, Stephanie S. M. H. Höhn, Aurelia R. Honerkamp-Smith, Julius B. Kirkegaard, Raymond E. Goldstein

**Affiliations:** 1 Department of Applied Mathematics and Theoretical Physics, Centre for Mathematical Sciences, University of Cambridge, Cambridge, United Kingdom; 2 Department of Physics, Lehigh University, Bethlehem, Pennsylvania, United States of America; The Beatson Institute for Cancer Research, Glasgow, United Kingdom of Great Britain and Northern Ireland

## Abstract

Variability is emerging as an integral part of development. It is therefore imperative to ask how to access the information contained in this variability. Yet most studies of development average their observations and, discarding the variability, seek to derive models, biological or physical, that explain these average observations. Here, we analyse this variability in a study of cell sheet folding in the green alga *Volvox*, whose spherical embryos turn themselves inside out in a process sharing invagination, expansion, involution, and peeling of a cell sheet with animal models of morphogenesis. We generalise our earlier, qualitative model of the initial stages of inversion by combining ideas from morphoelasticity and shell theory. Together with three-dimensional visualisations of inversion using light sheet microscopy, this yields a detailed, quantitative model of the entire inversion process. With this model, we show how the variability of inversion reveals that two separate, temporally uncoupled processes drive the initial invagination and subsequent expansion of the cell sheet. This implies a prototypical transition towards higher developmental complexity in the volvocine algae and provides proof of principle of analysing morphogenesis based on its variability.

## Author summary

Biological noise is unavoidable in—and even necessary for—development. Here, we ask whether this variability can teach us something about the process that underlies it. We show how to access the information hidden in the variability in an analysis of the variability of cell sheet folding in the green alga *Volvox globator*. Through a combination of light sheet microscopy and mathematical modelling, we show how the inversion process, by which the spherical embryos of *Volvox* turn themselves inside out, results from two separate mechanisms of bending and stretching (expansion and subsequent contraction). Our analysis therefore uncovers a prototypical transition of developmental complexity in *Volvox* and the related volvocine algae, from a morphogenetic process driven by a single mechanism to one driven by two separate mechanisms. This complements the similarly prototypical transition from one cell type to two cell types that has made the volvocine algae a model system for the evolution of multicellularity.

## Introduction

‘The phenomena are always the same, and this is what matters to us, but their variations, for the greater or for the lesser, are beyond count.’ Thus opined Xavier Bichat in the account of his investigations into life and death [1] and thereby spelt out how, to the present day, questions in developmental biology and cell sheet folding in particular are commonly approached: the vast majority of analyses average their experimental observations and seek to derive a model, biological or physical, that explains this average behaviour. In so doing, they discard the variability or deviations from average behaviour that are observed in experiments. A certain amount of noise is, however, unavoidable in biological systems; indeed, it may even be necessary for robust development, as demonstrated, for example, by Hong and colleagues [2], who showed that variability in cell growth is necessary for reproducible sepal size and shape in *Arabidopsis*. The natural question of how to use this variability to infer developmental mechanisms appears to lie in uncharted waters, however. This is the question that we explore in this paper to provide proof of principle of analysing cell sheet folding based on its variability.

Cell sheet folding pervades multicellular development, and its general principles have been established in a large body of previous work: local cellular changes can produce forces that are transmitted via cell–cell connections along the cell sheet and drive its global deformations [3, 4]. Simple events of cell sheet folding, such as ventral furrow formation in *Drosophila* [5, 6], can be driven primarily by cell shape changes. In more complex metazoan developmental processes—such as gastrulation [7, 8], optic cup formation [9, 10], neurulation [11, 12], and related processes in vivo [13] and in vitro [14]—the effect of such cell shape changes is overlaid by that of other cellular changes such as cell migration, cell intercalation, cell differentiation, and cell division. Owing to this complexity, and in spite of significant progress in identifying the molecular components involved, the correspondence between local cellular changes and global deformations of cell sheets remains poorly understood.

Biological analyses of morphogenesis are complemented, at a more physical level, by a whole host of mechanical models of morphogenesis. The first of these represented cells as discrete collections of springs and dashpots [15]; they were soon followed by elastic continuum models [16, 17]. Notable among this early modelling of morphogenesis is, for example, the work of Davidson and colleagues [18, 19], who combined models of several mechanisms of sea urchin gastrulation with measurements of mechanical properties to test the plausibility of these different mechanisms. These models heralded the emergence of a veritable plethora of mechanical modelling approaches over the subsequent decades [20], though the choice of model must ultimately be informed by the questions one seeks to answer [21]. More recent endeavours were directed at deriving models that can represent the chemical and mechanical contributions to morphogenesis and their interactions [22] and at establishing the continuum laws that govern these out-of-equilibrium processes [23].

However, all of this but emphasises a rather curious gap in the study of development: the importance of quantifying morphogenesis and its variability has been recognised [24, 25], but analyses of the variability of development have been few and far between. What experimental data there are on the variability of the mechanical properties of cell sheets suggest a large amount of variability ([26] and references therein). The variability of the cell sheet deformations during development is even more unexplored, and accounts of this variability—e.g., in the loach *Misgurnus fossilis* [27, 28]—have often been merely descriptive. In this paper, we present the first comprehensive analysis of this variability in cell sheet folding and the lessons that can be drawn from it.

**Fig 1:**
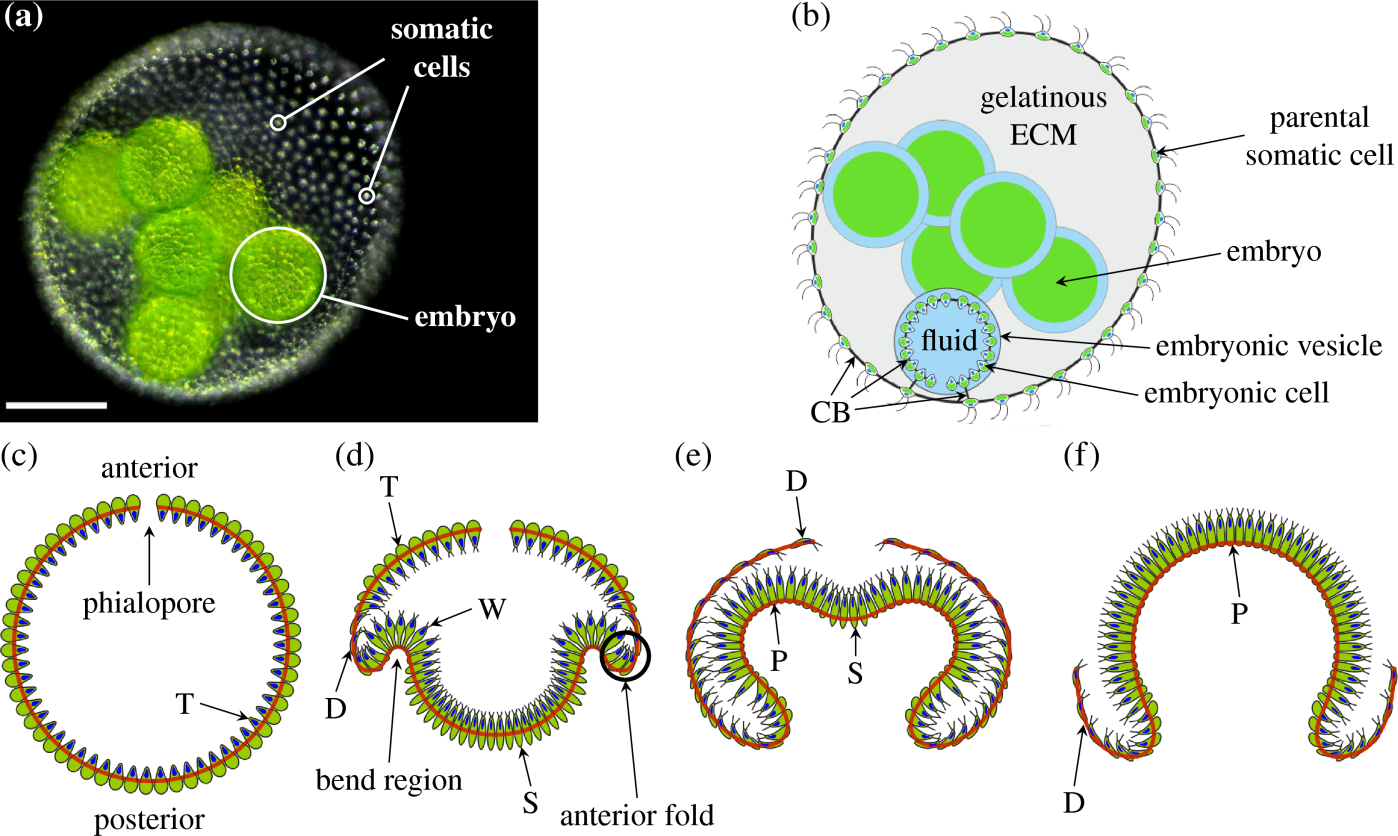
Morphology of and embryonic inversion in *V*. *globator*. (a) Adult spheroid with somatic cells and one embryo labelled. Scale bar: 50 μm. (b) Schematic drawing of *V*. *globator* parent spheroid with embryos. (c) Schematic drawing of *Volvox* embryo before inversion, with anterior and posterior poles and phialopore labelled. Cells are teardrop-shaped (labelled ‘T’). (d) *Volvox* invagination: the formation of wedge-shaped cells (labelled ‘W’) in the bend region initiates inversion. At the same time, cells in the posterior become spindle-shaped (labelled ‘S’), while cells close to the anterior fold (the region of increased positive curvature next to the bend region marked in the figure) become disc-shaped (labelled ‘D’). (e) At the end of posterior inversion, cells in the whole of the anterior hemisphere are disc-shaped, while cells in the bend region are pencil-shaped (labelled ‘P’). (f) As the anterior hemisphere peels over the inverted posterior, more and more cells become pencil-shaped. Red lines in panels c–f mark position of CBs. *Panel a adapted from [43] and panels c–f adapted from [44].* CB, cytoplasmic bridge; ECM, extracellular matrix.

The experimental system in which we explore these questions of variability is the multicellular green alga *Volvox* (Fig 1A), of which Julian Huxley said that ‘In some colony like *Volvox*, there once lay hidden the secret of the body and mind of [humans]’ [29]. Indeed, *Volvox* and the related volvocine algal genera have been recognised since the work of Weismann [30] as model organisms for the evolution of multicellularity [31–33], spawning more recent investigations of kindred questions in fluid dynamics and biological physics [34]. Being able to reproduce asexually, *Volvox* is perfectly suited for studying nongenetic sources of morphogenetic variation among genetically identical individuals. In the asexual life cycle, the cells of an adult *Volvox* spheroid (Fig 1B) are differentiated into biflagellate somatic cells and a small number of germ cells, or gonidia, that will form the next generation [31]. The somatic cells in the adult are embedded in a glycoprotein-rich extracellular matrix [35, 36]. The germ cells undergo several rounds of cell division, after which each embryo consists of several thousand cells arrayed to form a thin, spherical sheet confined to a fluid-filled vesicle. Cells are connected to their neighbours by cytoplasmic bridges (Fig 1B), thin membrane tubes resulting from incomplete cell division [37–39]. Those cell poles whence will emanate the flagella, however, point into the sphere at this stage, and so the embryos must turn themselves inside out through an opening at the anterior pole of the cell sheet (the phialopore) to enable motility and thus complete their embryonic development [31]. This process of inversion has become a model for the study of cell sheet deformations [40–42].

**Fig 2:**
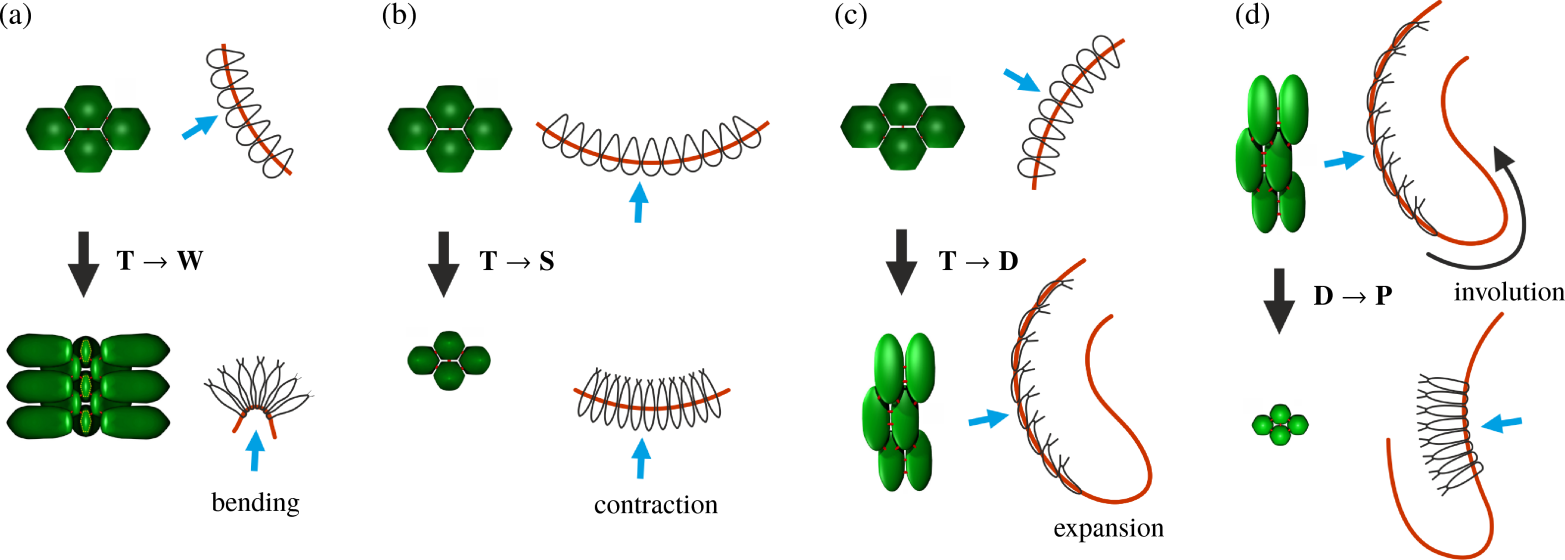
Cell shape changes in *V*. *globator*. Cell shape changes during inversion, associated with bending and stretching of the cell sheet, following [44]. Cell shape changes (black arrows) (a) from teardrop-shaped (labelled ‘T’) to wedge-shaped (labelled ‘W’) cells in combination with movement relative to the CBs, associated with invagination of the bend region; (b) from teardrop-shaped to spindle-shaped (labelled ‘S’) cells, associated with contraction of the posterior hemisphere; (c) from teardrop-shaped to disc-shaped (labelled ‘D’) cells, associated with expansion of the anterior hemisphere (before opening of the phialopore); and (d) from disc-shaped to pencil-shaped (labelled ‘P’) cells, associated with contraction of the anterior hemisphere and involution over the anterior fold. Red line: position of the CBs, blue arrows: direction of view of cell groups shown. CB, cytoplasmic bridge.

Inversion in *Volvox* [44, 45] and in related species [46–49] results from cell shape changes only, without the complicating additional processes found in metazoan development discussed above. This simplification facilitates the study of morphogenesis. While different species of *Volvox* have developed different ways of turning themselves inside out [46], here, we focus on the so-called type-B inversion arising, for example, in *V*. *globator* [44, 46, 50]. This shares features such as invagination and involution with developmental events in metazoans [51–53]. This inversion scenario is distinct from type-A inversion, in which four lips open at the anterior of the shell and peel back to achieve inversion [45]. This process is driven by a single wave of uniform cell shape changes moving from the anterior to the posterior pole of the embryo [45]. By contrast, type-B inversion involves different types of cell shape changes in different parts of the cell sheet [44], the coupling of which has remained unclear. This inversion begins with the appearance of a circular bend region at the equator of the embryo (Fig 1C and 1D, Fig 2A): cells there become wedge-shaped by developing narrow basal stalks [44]. At the same time, the cells move relative to the cytoplasmic bridges so as to be connected at their thin stalks, thus splaying the cells and bending the cell sheet [44]. Nishii and colleagues [54] showed that type-A inversion in *V*. *carteri* is arrested in the absence of analogous motion of cells relative to the cytoplasmic bridges. This relative motion is mediated by a motor protein, the kinesin InvA, associated to the microtubule cytoskeleton (S1 Fig); orthologues of InvA are found throughout the volvocine algae [32]. After invagination, the posterior hemisphere moves into the anterior (Fig 1E), the phialopore widens, and the anterior hemisphere moves over the subjacent posterior (Fig 1F) while 'rolling' over a second circular bend region, the anterior fold [44]. Additional cell shape changes (Fig 1D–1F, Fig 2B–2D) in the anterior and posterior hemispheres are implicated in the relative contraction and expansion of either hemisphere with respect to the other [44]. This plethora of cell shape changes is possible as embryonic *Volvox* cells do not have a cell wall [31]. It is not yet known what triggers the initial cell shape changes, what determines their location, and what kind of signal drives the propagation of waves of cell shape changes in *Volvox* embryos. In the present study, we show that even without this knowledge, we are able to infer information on local changes from the variability of global dynamic embryo shapes.

In a previous study [43], we combined light sheet microscopy and theory to analyse the early stages of inversion, showing that only a combination of active bending and active stretching (i.e., expansion or contraction) can account for the cell sheet deformations observed during invagination. The crucial role of active stretching was also highlighted by Nishii and Ogihara [55], who showed that type-A inversion in *V*. *carteri* cannot complete if actomyosin-mediated contraction is inhibited chemically. We later analysed the mechanics of this competition between bending and stretching in more detail [56]. The general question of how the different parts of a morphogenetic process relate to each other, however, remained unanswered in this system, too: are the different deformations of either hemisphere during type-B inversion coupled? What drives the 'peeling' of the anterior hemisphere?

The present analysis addresses these questions and naturally divides into three parts: we begin by deriving an average sequence of *Volvox* inversion and quantifying its variability. This consensus inversion sequence serves as a template for the mathematical analysis in the second part of the paper: building on our earlier, qualitative model of the early stages of inversion and combining ideas from morphoelasticity [57] and shell theories [58, 59], we derive a detailed, quantitative description of the entire process of inversion. In so doing, we show for the first time how detailed information on the underlying cellular changes can be deduced from deformations at the tissue level. In the third and final part of the paper, we compare the experimental distribution of variability to simulated distributions based on perturbations of the local active deformations in the model. We thus infer how the observed distribution of variability in the embryo shapes arises, and we find that inversion is driven by two separate, temporally uncoupled processes. This provides proof of principle of using developmental variability to infer developmental mechanisms and mechanics.

## Results

**Fig 3:**
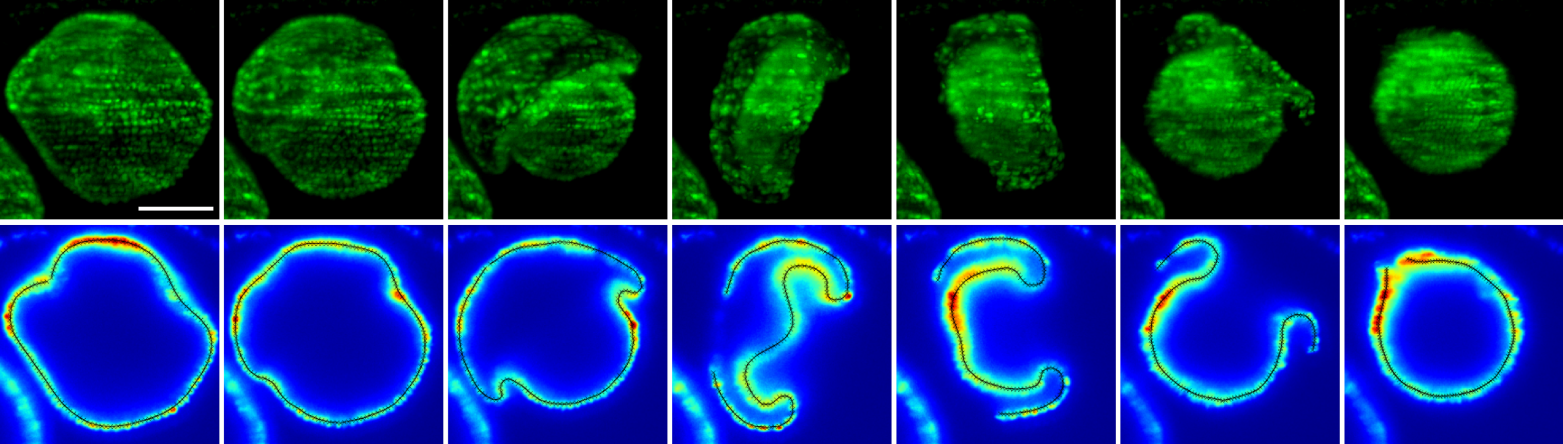
Experimental visualisation of type-B inversion. Inverting *V*. *globator* embryo visualised by SPIM of chlorophyll autofluorescence. Top row: maximum-intensity projection of z-stacks. Bottom row: tracing of midsagittal cross-sections (Materials and methods); the colour scheme indicates image intensity. Scale bar: 50 μm. SPIM, selective plane illumination microscopy.

We acquired three-dimensional time-lapse visualisations of inverting *V*. *globator* embryos (Fig 3 and Materials and methods) using a selective plane illumination microscopy (SPIM) setup (Materials and methods) based on the OpenSPIM system [60]. Data were recorded for 13 parent spheroids containing, on average, 6 embryos. Summary statistics for 33 embryos were obtained from the recorded z-stacks (S1 Text). These statistics revealed considerable variability, even between embryos from the same parent spheroid (S1 Text). For a more quantitative analysis of inversion, embryo outlines were traced on midsagittal sections of 11 of the recorded inversion processes, selected for optimal image quality (Materials and methods).

We have previously described type-B inversion in terms of a set of geometric descriptors; we extend this description in S1 Text. To quantify the variability of inversion, we must, however, obtain a consensus inversion sequence; to do so, we must begin by defining an average of embryonic shapes. This average must scale out each of the following types of variability that can arise during inversion: (1) different embryos may have different sizes, (2) different embryos may take different amounts of time to reach the same stage of inversion because the cell shape changes driving inversion may arise at different times, and (3) these cell shape changes may arise at different positions in the cell sheet. Our analysis will be based on this consensus sequence, since the amount of variability already revealed in S1 Text implies that an analysis based on unscaled embryo shapes in absolute real time cannot be meaningful.

### The average inversion and its local variability

**Fig 4:**
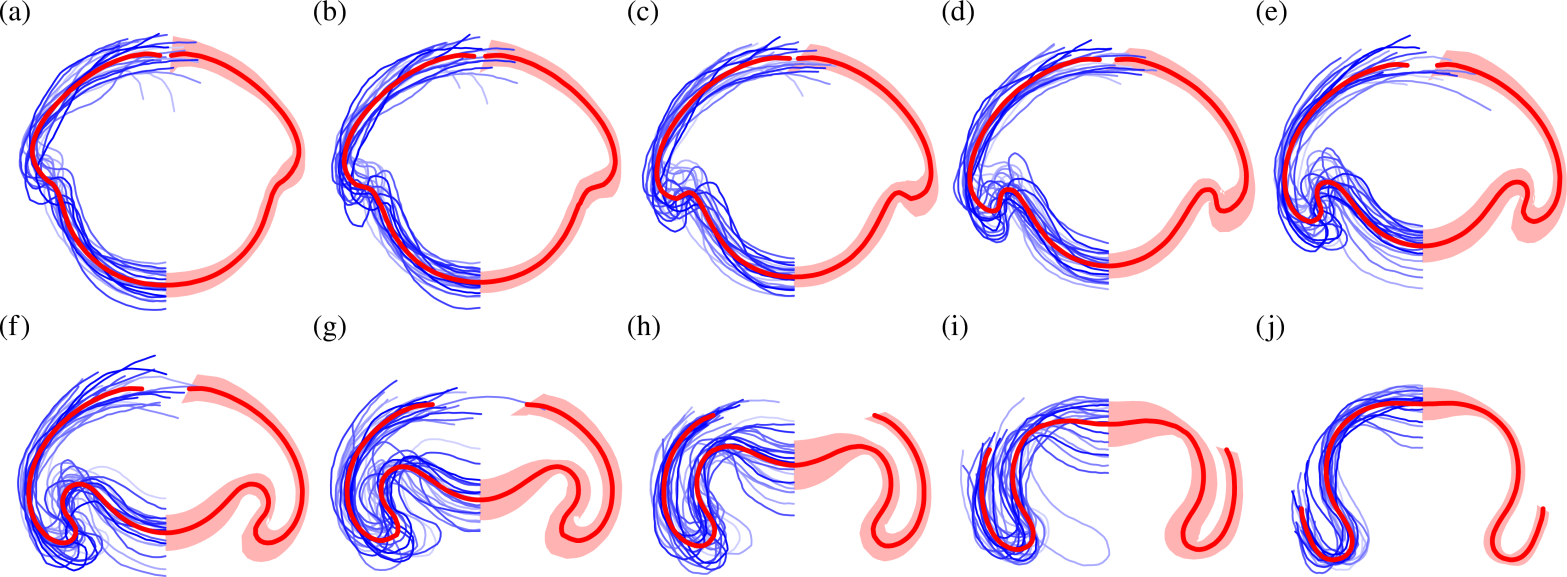
Average stages of inversion. Average shapes of *Volvox* embryos for 10 stages of inversion (red lines), obtained from *N* = 22 overlaid and scaled embryo halves (lines in shades of blue on the left) and corresponding standard deviation shapes (shaded areas on the right). Note the increased standard deviation in the anterior fold compared to the bend region during invagination in panels c–e. See S1 Data for numerical values.

To define an average inversion sequence and analyse its mechanics, we compare the local geometry of the traced curves. The rather philosophical question of how to define an appropriate metric for this kind of comparison goes back at least to the work of D'Arcy Thompson [61] and has no unique answer. Thompson showed, for example, how the outlines of fish of different species could be mapped onto one another by dilations, shears, and compositions thereof. Our averaging approach must allow for the different types of variability that arise in *Volvox* inversion (as discussed in the previous paragraph), while recognising that the posterior poles and the rims of the phialopores of the different embryos must correspond to each other. Our approach is therefore based on minimising the euclidean distance between individual embryo shapes and their averages, with alignments obtained using dynamic warping of shapes (Materials and methods). Results are shown in Fig 4.

Averaging approaches that do not consider both stretching in time of individual inversions and local stretching of corresponding points of individual shapes tend to give unsatisfactory results: the simplest averaging approach is to align the inversion sequences by a single time point—for example, when the posterior-to-bend distance reaches half of its initial value (Materials and methods and S2 Fig). The absence of time stretching, however, means that large variations arise at later stages of inversion. (Given the dramatic embryonic shape changes during inversion, it is not surprising that there should be no single parameter that could be used to align inversions of different embryos.) A better alignment is obtained if we allow stretching in time (Materials and methods and S3 Fig), but this method, without local stretching of individual shapes relative to each other, produces unrepresentative kinks in the bend region of the average shapes (S3 Fig).

**Fig 5:**
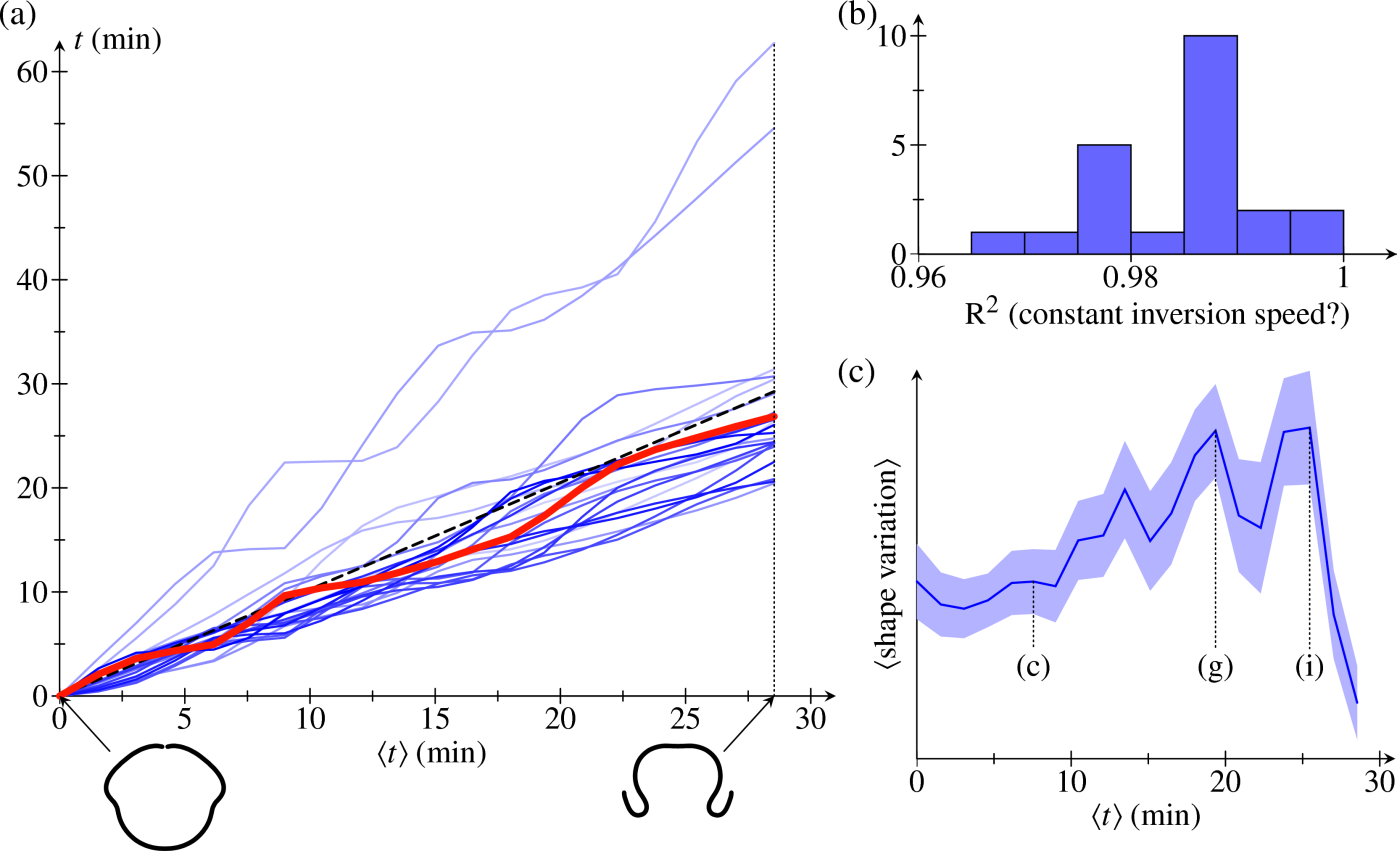
Alignment statistics. (a) Timepoints *t* for *N* = 22 embryo halves (relative to first fitted timepoint) plotted against the mean values ⟨*t*⟩ of these times. Red line: time evolution illustrating the 'stop-and-go' progression of inversion. Insets: average embryo shapes at earliest and latest fitted times. (b) Histogram of R^2^ statistic for straight-line fits of the time evolutions in the first panel, corresponding to a constant inversion speed. (c) Mean shape variation (in arbitrary units), and standard errors thereof, against mean time ⟨*t*⟩. Corresponding panels in Fig 4 are marked for some data points. See S1 Data for numerical values.

To quantify the time course of individual inversions further, we shift the time coordinate of each embryo half so that *t* = 0 is the time when it reaches the first fitted stage. We then define the average time ⟨*t*⟩ to be the average, over all embryo halves, of these shifted times. Plotting the time course of individual inversions in the resulting (⟨*t*⟩,*t*) diagram (Fig 5A), we observe that different stages of inversion take different times in different embryos, with some embryos seeming to linger in certain stages. Nonetheless, despite this 'stop-and-go' behaviour, each time course is well approximated by a straight line in the (⟨*t*⟩,*t*) diagram (Fig 5B), which signifies that inversion proceeds at constant speed in all embryos.

To analyse the local variations of the embryo shapes, we define, at each point of the average shapes, a covariance ellipse. The curves that are parallel to the average shape and tangent to the covariance ellipse define what we shall term the standard deviation shape. These standard deviation shapes measure the variability of the average shapes and are shown in Fig 4. The variations they represent naturally divide into two components: first, those variations that are parallel to the average shape and, second, those perpendicular to the average shape. The former represent mere local stretches of the average shapes, while the latter correspond to actual variations of the shapes; we shall therefore refer to the thickness of the standard deviation shapes as 'shape variation' in what follows. We report the mean shape variation and its standard error in Fig 5C. This plot shows that the mean shape variation reaches a maximal value around the stages in Fig 4G–4I: different embryos start from the same shape and reach the same inverted shape after inversion (up to a scaling) but may take different inversion paths. Plotting the mean shape variation for different averaging methods (S4 Fig), we confirm that the present averaging method yields a better alignment than the alternative methods discussed earlier.

It is intriguing, however, to note the spatial structure of the local shape variations. In particular, during the early stages of posterior inversion (Fig 4D–4F), the shape variation is smaller in the active bend region than in the adjacent anterior fold (Fig 1E, the second bend region of increased positive curvature). As the phialopore opens, and the anterior begins to peel back over the partially inverted posterior (Fig 4H), the relative shape variation becomes smaller in the anterior fold. The initially small variation in the bend region is especially intriguing, since this is where cells become wedge-shaped to drive invagination, while the anterior fold bends passively [43]. In other words, the shape variation is reduced in the part of the cell sheet where the active cell shape changes that drive invagination arise. If there were no variability in the cell shape changes, then inversion could not fail. This correspondence therefore characterises what one might term, from a teleological point of view, a 'good' inversion. We shall focus on a less exalted question, the answer to which will be falsifiable, however: how is this spatial structure of the variability related to the mechanics of inversion? Before we can address this question, we need to analyse the mechanics of inversion in some more detail.

### A quantitative elastic model of inversion

**Fig 6:**
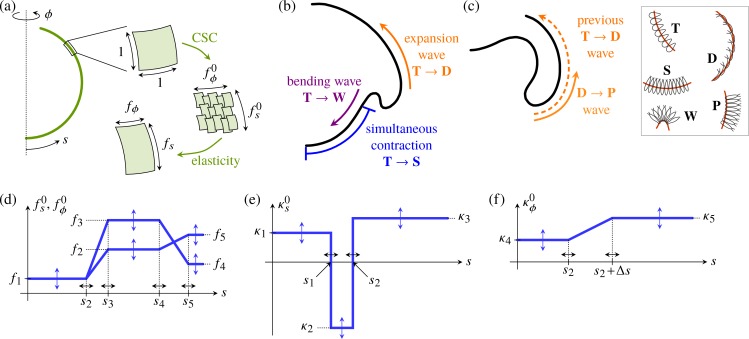
Mechanics of inversion: Elastic model and open questions. (a) Cell shape changes (labelled ‘CSCs’) endow an axisymmetric elastic shell with intrinsic meridional (*s*) and circumferential (*ϕ*) stretches $f_{s}^{0},f_{\phi }^{0}$. Since these are, in general, incompatible with the global geometry of the shell, it deforms elastically to adopt stretches *f*_*s*_,*f*_*ϕ*_. Analogously, the intrinsic curvatures $\kappa_{s}^{0},\kappa_{\phi }^{0}$ of the shell differ from its actual curvatures *κ*_*s*_,*κ*_*ϕ*_. Detailed mathematical derivations are provided in the Materials and methods section. (b) Inversion mechanics: our earlier work [43] revealed that active bending, contraction, and expansion are necessary for the early stages of inversion, but did not resolve the coupling of the waves of bending and expansion observed in [44]. (c) The peeling of the anterior hemisphere during the later stages of inversion is associated with another wave of cell shape changes [44], but the mechanical basis for the peeling has remained unclear. Cell shape changes **A** → **B** between cell types **A**,**B** are indicated in panels b,c, with the legend on the right of panel c recalling the definitions of cell types of Fig 2. Red lines in insets mark the position of cytoplasmic bridges. (d) Piecewise constant or linear functional form of the intrinsic stretches $f_{s}^{0},f_{\phi }^{0}$, plotted against the arclength *s* of the undeformed shell. (e) Functional form of the meridional intrinsic curvature $\kappa_{s}^{0}$. (f) Functional form of the circumferential intrinsic curvature $\kappa_{\phi }^{0}$. Labels in panels d, e, and f define the 15 parameters *f*_1_,…,*f*_5_, *κ*_1_,…,*κ*_5_, *s*_1_ <…< *s*_5_ discussed in the text. The values of these parameters depend on the inversion stage and are obtained numerically by a fitting algorithm comparing experimental and numerical embryo shapes.

The second step of our analysis is to derive a quantitative theoretical model of inversion. We have previously described the early stages of inversion using a qualitative mathematical model [43] in which cell shape changes appear as local variations of the intrinsic (meridional and circumferential) curvatures $\kappa_{s}^{0},\kappa_{\phi }^{0}$ and stretches $f_{s}^{0},f_{\phi }^{0}$ of an elastic shell (Fig 6A). Open, one-dimensional elastic filaments can simply adopt a shape in which the curvature and stretch are everywhere equal to their intrinsic values, but two-dimensional elastic shells cannot, in general, do this: the intrinsic curvatures and stretches may not be compatible with the global geometry, causing the shell to deform elastically and adopt actual (meridional and circumferential) curvatures *κ*_*s*_,*κ*_*ϕ*_ and stretches *f*_*s*_,*f*_*ϕ*_ different from the imposed intrinsic curvatures and stretches (Fig 6A). A more technical discussion of these issues, couched in the language of differential geometry, is provided in [57].

Our previous model [43] revealed that active bending, active contraction, and active expansion are necessary for the early stages of inversion (Fig 6C). The relation between these processes remained unclear, however, and the model could not describe the large deformations during later stages of inversion (Fig 6D). Indeed, that model was derived under the assumption of small strains. While the elastic strains are small indeed (since the metric tensor, which describes the deformed shape, is close to the intrinsic tensor defined by the cell shape changes), the geometric strains are large: both the metric tensor of the deformed shell and the intrinsic tensor differ considerably from the metric tensor of the undeformed sphere. We must therefore generalise our previous mathematical model by combining ideas from morphoelasticity and shell theories (Materials and methods) in order to obtain a quantitative description of the entire inversion process.

The cell shape changes (Fig 6B and 6C; see also Fig 1C–1F and Fig 2) observed previously in [44] suggest simple functional forms of the intrinsic stretches and curvatures defined in terms of 15 parameters (Fig 6D–6F) that vary over the course of inversion: the parameters *f*_1_,…,*f*_5_ and *κ*_1_,…,*κ*_5_ encode the magnitudes of the intrinsic stretches and curvatures of the different cell types that arise in different positions of the cell sheet at different times during inversion, while the parameters *s*_1_ <…< *s*_5_ encode the arclength positions of the transitions between cell types. These 15 parameters allow for a minimal representation of the cell shape changes [44] and ensuing variations of the intrinsic stretches and curvatures:

- The intrinsic stretches $f_{s}^{0},f_{\phi }^{0}$ vary in the both hemispheres (Fig 6D): in the posterior hemisphere, the initially teardrop-shaped cells thin into spindle-shaped cells (Fig 6D, Fig 1C and 1D, Fig 2B), while, in the anterior hemisphere, they flatten into disc-shaped cells (Fig 6B, Fig 1D and 1E, Fig 2C). While the evolution towards spindle-shaped cells appears to occur at the same time all over the posterior hemisphere, the data from thin sections [44] suggest that the transition to disc-shaped cells starts at the bend region and progresses towards the phialopore (Fig 6B, Fig 1D and 1E). Moreover, the spindle-shaped cells are isotropic, $f_{s}^{0}\approx f_{\phi }^{0}=f_{1}$, while the disc-shaped cells are markedly anisotropic (Fig 2C): next to the bend region, the long axis of their elliptical cross-section is the meridional one ($f_{\phi }^{0}=f_{2}<f_{3}=f_{s}^{0}$); next to the phialopore, it is the circumferential axis ($f_{s}^{0}=f_{4}<f_{5}=f_{\phi }^{0}$, with *f*_3_ > *f*_4_,*f*_5_ > *f*_2_). For simplicity, we impose the same values of intrinsic stretches for the spindle-shaped cells and the wedge-shaped cells in the bend region.
- The meridional intrinsic curvature $\kappa_{s}^{0}$ (Fig 6E) is expected to vary most drastically in the region where wedge-shaped cells with thin ends and, later, pencil-shaped cells form (Fig 6B and 6C, Fig 1D, Fig 2A and 2D). Because of the motion of cytoplasmic bridges relative to the cells, some additional yet slighter variation may be expected, requiring three parameters *κ*_1_,*κ*_2_,*κ*_3_.
- The variations of the circumferential intrinsic curvature $\kappa_{\phi }^{0}$ are less clear: on the one hand, $\kappa_{\phi }^{0}$ does not vary as drastically as the meridional one, because of the anisotropy of the wedge-shaped cells [43]. On the other hand, some variation of the circumferential intrinsic curvature may be expected because of the motion of cytoplasmic bridges (Fig 6F). We impose a continuous functional form for $\kappa_{\phi }^{0}$, regularising a step function between two values *κ*_4_,*κ*_5_ over a distance Δ*s* in arclength (Fig 6F).

**Fig 7:**
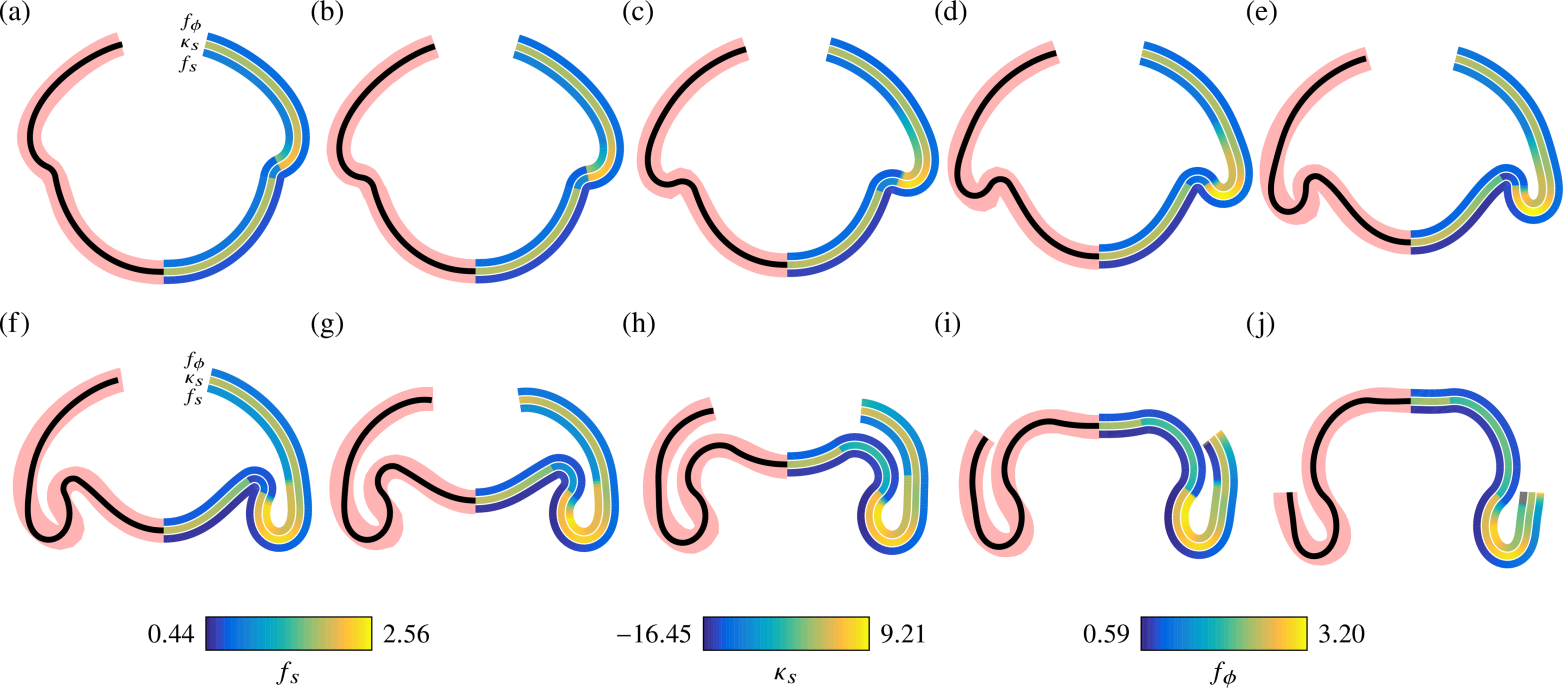
Average embryo shapes reproduced by the elastic model. In each panel, the left half shows average shapes from Fig 4 (thick red line) and corresponding fits (black line) from the elastic model for different stages of inversion. The right half shows colour-coded representations of the meridional curvature *κ*_*s*_ and stretches *f*_*s*_ (meridional) and *f*_*ϕ*_ (circumferential) in the fitted shapes. See S1 Data for numerical values.

We proceed to fit the generalised elastic model to the experimental average embryo shapes (Materials and methods). The fitting algorithm compares experimental and numerical embryo shapes to obtain values of the 15 parameters described above for each stage of inversion. In the model, we impose a larger extent of the phialopore than in the biological system, in which the phialopore is initially very small (Fig 4A). This is an important simplification to deal with the discrete nature of the few cells that meet up at the phialopore. In spite of this simplification, the model captures the various stages of inversion (Fig 7); fitted numerical values of the 15 parameters are given in S1 Data. This supports our interpretation of the observed cell shape changes (Fig 6B and 6C, Fig 2) and their functions.

#### Elastic model predicts stretches in agreement with cell size measurements

To validate the model, we show that the parameter values obtained by the fitting algorithm are consistent with what we know about the underlying cell shape changes. To this end, we relate the values of *f*_*s*_,*f*_*ϕ*_ in the fitted shapes, shown in Fig 7, to the measurements of individual cells in fixed embryos in [44]: before inversion starts, the cells are teardrop-shaped and measure 3–5 μm in the plane of the cell sheet. As invagination starts, the cells in the posterior hemisphere become spindle-shaped, measuring 2–3 μm. This suggests values *f*_*s*_,*f*_*ϕ*_ ≈ 0.6 − 0.66 in the posterior hemisphere during invagination, in agreement with the fitted data (Fig 7D). At later stages of inversion, the cells in the bend region become pencil-shaped, measuring 1.5–2 μm in the meridional direction, suggesting smaller values *f*_*s*_ ≈ 0.4 − 0.5 there, again in agreement with the fitted data (Fig 7H). The large stretches *f*_*s*_ > 2 seen in the anterior fold during inversion of the posterior hemisphere (Fig 7F) cannot be accounted for by the disc-shaped cells in the anterior (which only measure 4–6 μm in the meridional direction). While examination of the thin sections of [44] does suggest, in qualitative agreement with the fits, that the largest meridional stretches arise in the anterior fold, the fact that the model overestimates the actual values of these stretches may stem from the simplified modelling of the phialopore. Further, at the very latest stages of inversion (Fig 7J), the fitted shapes suggest very small values *f*_*s*_ < 0.3 and corresponding values *f*_*ϕ*_ > 3 that are not borne out by the cell measurements.

Comparing the observed cell shape changes and the fitted values of the intrinsic curvatures and stretches in this way is an important consistency check on our solution of the fitting problem—i.e., the inverse problem of inferring the intrinsic parameters from the experimental averages. Indeed, the 'exact' inverse problem of inferring the intrinsic parameters from a deformed shape produced by the model does not necessarily have a unique solution, as we have previously illustrated for simple deformations [56].

#### Posterior inversion results from a uniformly expanding wave of cell shape changes to wedge shapes

**Fig 8:**
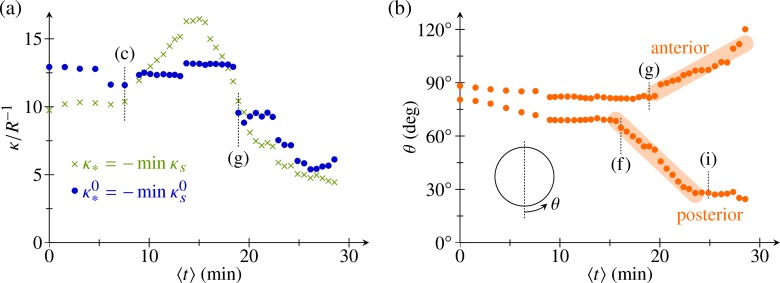
Analysis of fitted parameters. (a) Plot of most negative values of the intrinsic and actual meridional curvatures, $\kappa_{*}^{0}=-\min\kappa_{s}^{0}$ and κ_*_ = −min *κ*_*s*_ against mean time ⟨*t*⟩. (b) Positions of posterior and anterior limits of the bend region relative to the undeformed sphere, plotted against mean time ⟨*t*⟩. Thick lines indicate straight line fits. Corresponding panels in Fig 7 are marked for some data points. See S1 Data for numerical values.

Having thus validated the model, we are in a position to use this detailed theoretical description to gain additional information about the underlying cell shape changes. We begin by addressing a cell shape conundrum: during invagination, the curvature in the bend region increases (Fig 7), yet Höhn and Hallmann [44] reported similar wedge-shaped cells in the bend region at early and late invagination stages, although the number of wedge-shaped cells in the bend region increases as invagination progresses [44]. The fitted parameters indeed suggest a constant value of the intrinsic curvature at early stages of inversion, while the actual curvature in the bend region increases (Fig 8A). This serves to illustrate that the intrinsic parameters cannot simply be read off the deformed shapes and confirms that there is but a single type of cell shape change, expanding in a wave to encompass more cells and thus driving invagination. It is only at later stages of inversion, when the wedge-shaped cells in the bend region become pencil-shaped [44], that both the intrinsic curvature and the actual curvature in the bend region decrease (Fig 8A).

The fitted shapes also yield the posterior and anterior limits of the bend region (Fig 8B)—i.e., the original positions, relative to the undeformed sphere, of the corresponding cells. Because of the varying spatial stretches of the shell, these positions again cannot simply be read off the deformed shapes but must be inferred from the fits. The fitted data suggest that invagination results from an intrinsic bend region of constant width, complemented by other cell shape changes (Fig 1D, Fig 2B and 2C). The region of wedge-shaped cells (and, by implication, of negative intrinsic curvature) starts to expand into the posterior at constant speed (i.e., at a constant number of cell shape changes per unit of time) between the stages in Fig 7E and 7F. Anterior inversion starts about 5 min later when this region begins to expand into the anterior just after the stage in Fig 7G. Since we cannot currently visualise the shapes of single cells in vivo, this information about the timing of the cell shape changes can indeed only be inferred from the detailed analysis of the model.

#### Phialopore opening is associated with cell rearrangement

**Fig 9:**
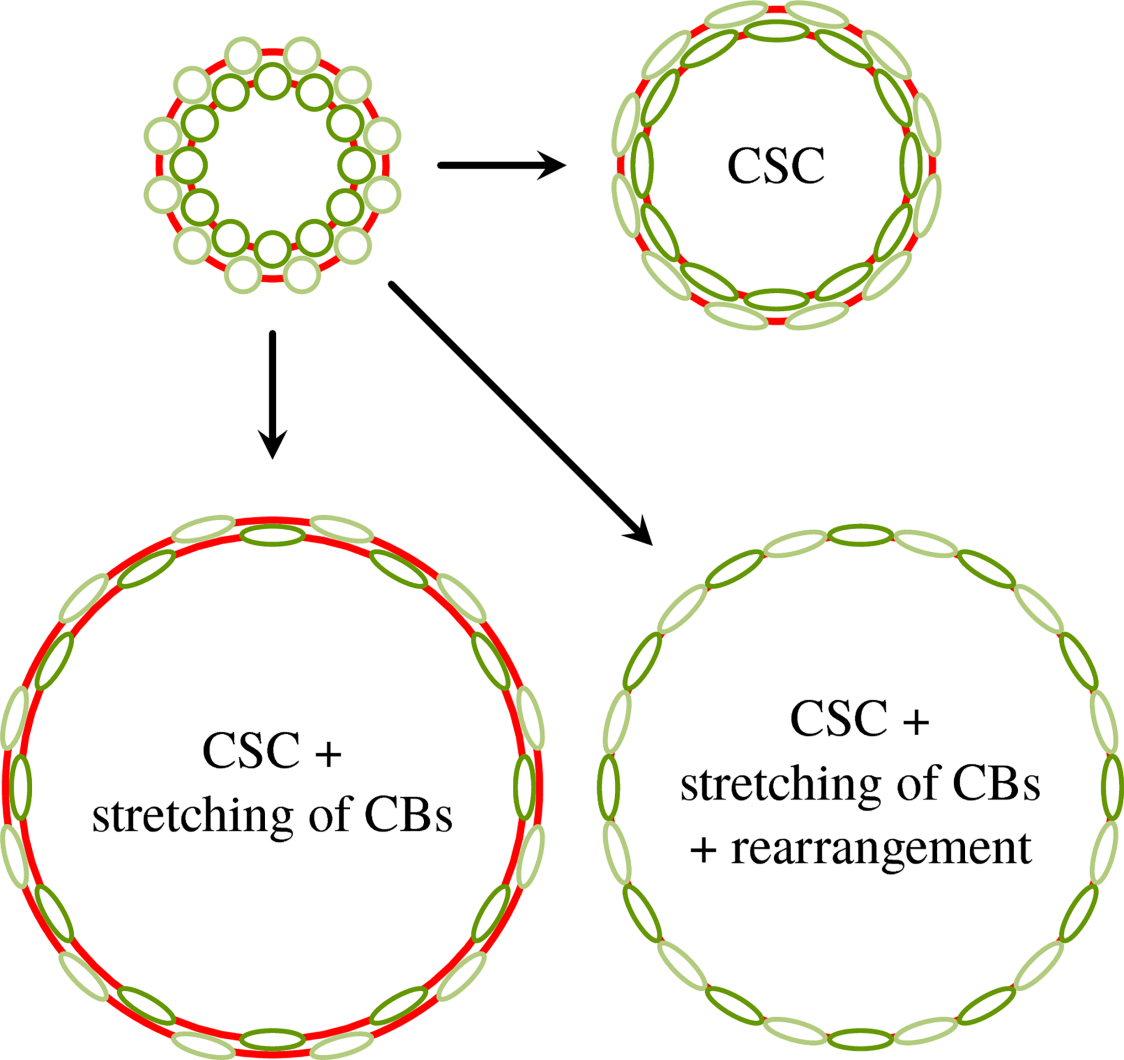
Scenarios of phialopore opening. Possible scenarios of phialopore widening by cell shape changes (labelled ‘CSCs’), stretching of cytoplasmic bridges (labelled ‘CBs’), and cell rearrangements. Views of cells are from the top, parallel to the embryo axis and onto the phialopore. Red lines represent cytoplasmic bridges; fainter colours signify cells further away from the phialopore in the original configuration.

**Fig 10:**
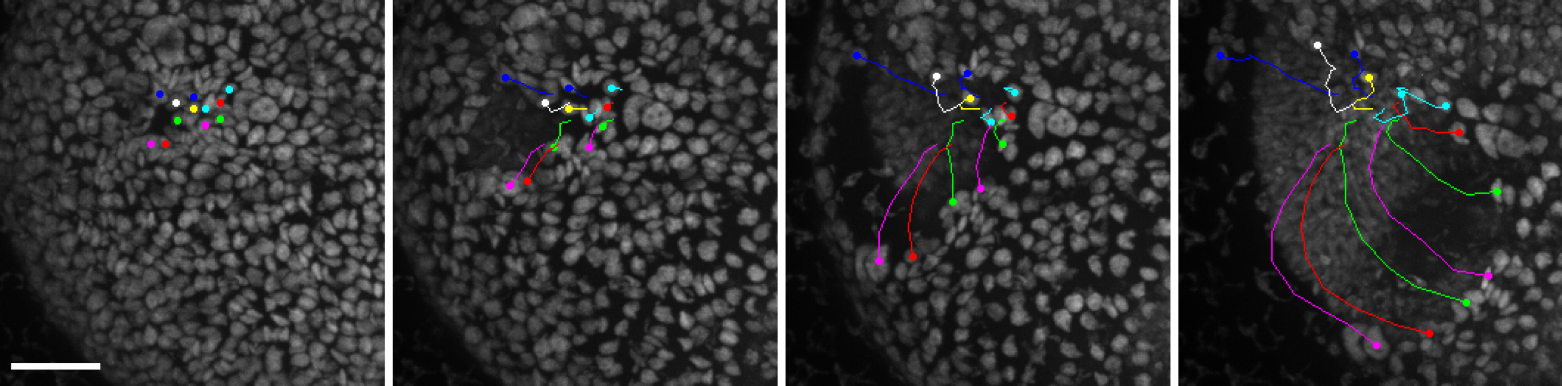
Cell rearrangement during phialopore opening. Rearrangement of cells surrounding the phialopore during phialopore opening. Images obtained from confocal laser scanning microscopy of chlorophyll autofluorescence and manual tracing of selected cells (Materials and methods). Scale bar: 20 μm.

At this stage, to complete our analysis of the model, we briefly interrupt the flow of the narrative to understand why, as discussed above, the fitted values of the stretches at the phialopore are at odds with the observed cell shape changes. We must therefore analyse the opening of the phialopore in more detail. The observations of [44] show that the cytoplasmic bridges stretch considerably, to many times their initial length, as the phialopore opens. Circumferential elongation of cells as a means to increase effective radius was discussed in some detail in [62] but is not sufficient to explain the circumferential stretches observed at the phialopore. Additional elongation of cytoplasmic bridges as a means to further increase the effective radius (Fig 9) may suffice to produce the large circumferential stretches but does not explain the small values of meridional stretch at the phialopore in the fitted shapes. For this reason, we additionally imaged the opening of the phialopore using confocal laser scanning microscopy (Materials and methods) to resolve single cells close to the phialopore (Fig 10 and S2 Video).

The data reveal that cells rearrange near the phialopore, indicating the possibility of viscoelastic behaviour near the edge of the cell sheet and suggesting an additional mechanism to stretch the phialopore sufficiently for the anterior to be able to peel over the inverted posterior (Fig 9). Fig 10 and S2 Video show how initially only a small number of cells form a ring at the anterior pole. When the phialopore widens, cells that were initially located away from this initial ring come to be positioned at the rim of the phialopore. It is unclear whether the cytoplasmic bridges between these cells stretch or break or whether these cells were not connected by cytoplasmic bridges in the first place. While such cell rearrangement is beyond the scope of the current model, it is nevertheless captured qualitatively by the small values of *f*_*s*_ near the phialopore. Kelland [63] observed elongation of cytoplasmic bridges near the phialopore of *V*. *aureus*, but not in small fragments of broken-up embryos, and concluded that the elongation of cytoplasmic bridges was the result of passive mechanical forces. By contrast, in our model, the opening of the phialopore is the result of active cell shape changes there. This discrepancy may herald a breakdown of the approximations made to represent the phialopore. The data also hint that there may be a different mechanical contribution at later stages of inversion (Fig 4I), during which the rim of the phialopore may be in contact with the inverted posterior. Since the model does not resolve the rim of the phialopore in the first place, we do not pursue this further here. For completeness of the mechanical analysis, we analyse such a contact configuration in S2 Text, in which we also discuss a toy problem to highlight the intricate interplay of mechanics and geometry in the contact configuration.

### Local shape variations reveal inversion mechanisms

At this stage, we are finally set up to relate the spatial structure of the shape variations to the mechanisms and mechanics of inversion. This structure of the local shape variations results from variations of the underlying cell shape changes, via the mechanics of inversion, and from geometric effects associated with averaging the shapes.

Some of the structure observed in Fig 4 is clearly geometric: since the shapes are aligned so that the positions of their centres of mass along the axis coincide (Materials and methods), the shape variations accumulate and are thus expected to, e.g., increase in the anterior hemisphere, towards the phialopore, as at the stage in Fig 4C. At the same stage, however, the shape variation is smaller in the bend region than in the adjacent anterior fold. Both of these regions are, however, close to the centre of mass, and so we do not expect this difference to arise from mere geometric accumulation of shape variations. We must therefore ask: can this global structure arise purely mechanically (i.e., from a uniform variability of the local parameters implementing the changes of intrinsic curvatures and stretches so that each parameter varies by the same relative amount), but possibly as a statistical fluke, or must there be some regulation (i.e., nonuniform variability of these local intrinsic parameters)?

#### Uniform parameter variability cannot account for the observed shape variability

**Fig 11:**
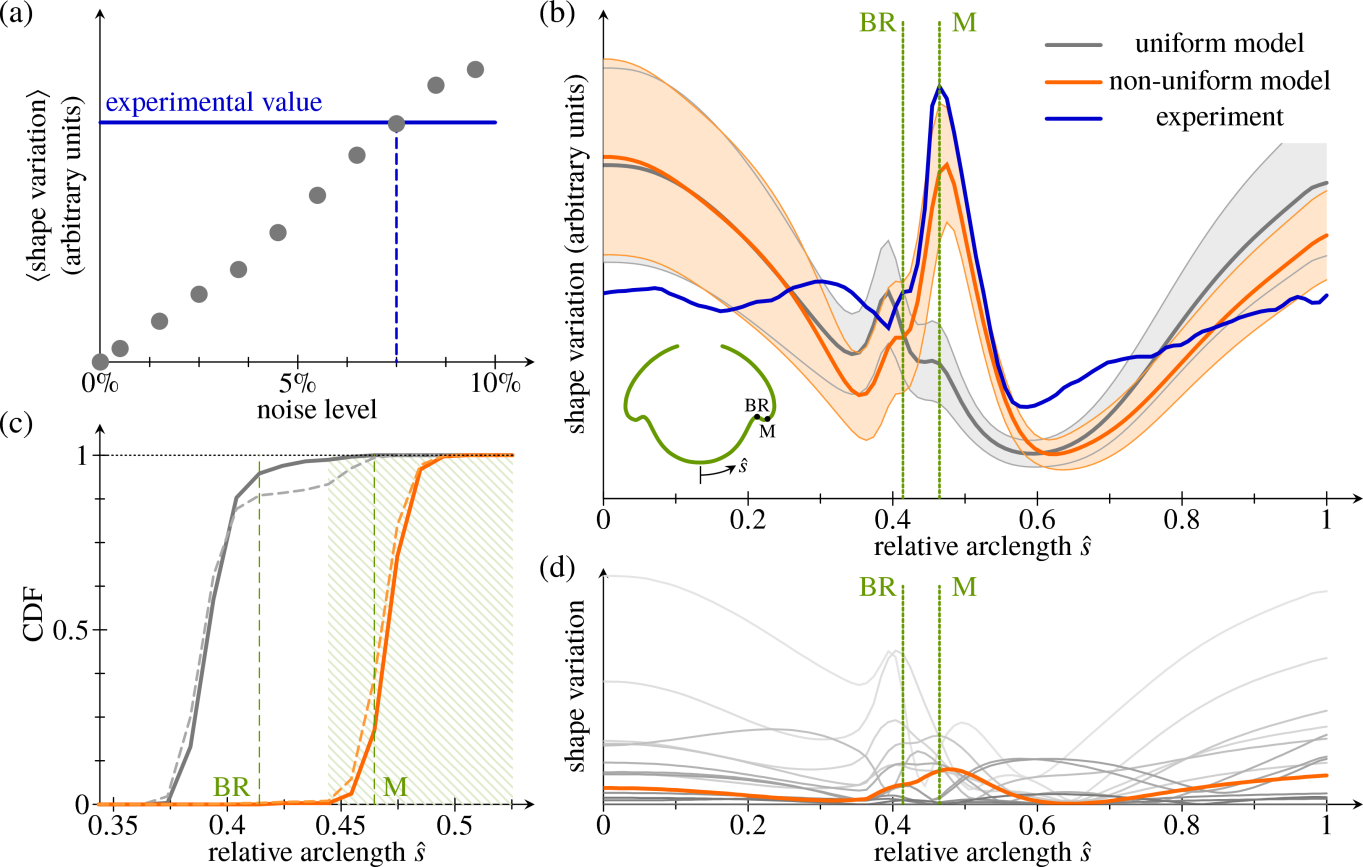
Analysis of shape variations. (a) Mean shape variation (in arbitrary units) against magnitude of uniform perturbations to the fitted shape of the stage in Fig 4C. Each data point was obtained from 1,000 perturbations of the fitted shape. Horizontal line: mean shape variation obtained from the experimental data. (b) Magnitude of shape variations against (deformed) arclength. Thick blue line: experimental average from *N* = 22 embryo halves. Grey line and grey shaded area: average and standard deviation of 1,000 samples of *N* = 22 perturbations each under the uniform model. Orange line and orange shaded area: corresponding plot with increased variability in the anterior fold. Inset: average shape of Fig 4C, with BR and position of the maximum (labelled ‘M’) of the experimental shape variation marked; these positions are marked by dotted lines in the main diagram. (c) CDF of the positions of the peak (local maximum) of shape variation under the uniform (grey lines) and modified (orange lines) models, with positions of the BR and of the maximum (labelled ‘M’) of the experimental distribution from panel b labelled. Dashed lines show distributions from all random perturbations; solid lines show those from shape variations with a single local maximum. A maximum is considered to lie in the anterior fold if it falls within the hatched region, which is used for the statistical estimates in the Materials and methods section. (d) Magnitude of shape variations induced by varying individual parameters (grey lines); each line is average of 250 samples of *N* = 22 perturbations each. Only increased variability of the stretching in the anterior fold (orange line) produces a single narrow peak of variability close to the experimental maximum. See S1 Data for numerical values. BR, bend region; CDF, cumulative distribution function.

To test whether the increased variability in the anterior fold compared to the bend region can be a result of uniformly distributed variations of the intrinsic parameters, we randomly perturb the fitted intrinsic parameters of the inversion stage in Fig 4C (Materials and methods). We begin by estimating the relative size of the perturbations (the 'noise level') in the experimental data. To do so, we compare the observed mean shape variation computed from the *N* = 22 embryo halves in Fig 4C to the mean shape variation estimated, for different noise levels, from 1,000 random perturbations of the fitted intrinsic parameters. Thus, we roughly estimate a noise level of 7.5% (Fig 11A).

With this noise level, we obtain 1,000 samples of *N* = 22 perturbations to the fitted intrinsic parameters each, and we compute their averages in the same way as for the experimental samples. (Raw data and statistics for all the random perturbations discussed in this section are given in S1 and S2 Data). While these samples qualitatively capture the spatial structure of the shape variation, they overestimate the shape variation at the poles (Fig 11B). More strikingly, they feature a local maximum of the shape variation in the bend region rather than in the anterior fold; additionally, this maximum is much less pronounced than in the experimental data. From the sample distribution of the position of these local maxima (Fig 11C), it is clear that the experimental distribution with the local maximum in the anterior fold is very unlikely to arise under these uniform perturbations. We make this statement more precise statistically in the Materials and methods section. We conclude that uniform variability of the underlying cell shape changes cannot explain the observed shape variability.

#### Expansion of the anterior hemisphere is temporally uncoupled from posterior inversion

To explain the observed structure of the shape variation, we must therefore allow for a nonuniform parameter distribution. Analysing the shape variation induced by varying each parameter individually at a noise level of 7.5%, we find that only the meridional stretch in the anterior fold contributes a single, narrow maximum of shape variation there (Fig 11D). We therefore allow more variability in the meridional stretch in the anterior fold (with a noise level of 60%, compared to 4.5% for the remaining parameters to reproduce the mean shape variation). The resulting distribution is consistent with the experimentally observed position of the local maximum of shape variation in the anterior fold (Fig 11B and 11C). While still overestimating the variability near the posterior pole, this modified distribution of the parameter variability captures the magnitude of the variability in the anterior fold much better than the original one.

Thus, at this early stage of inversion (Fig 4C), the observed embryo shapes are consistent with an increased variation of the intrinsic meridional stretch in the anterior fold. At the cellular level, these variations are associated with the formation of disc-shaped cells there (Fig 1D, Fig 2C). This increased variability of the meridional stretch could either be imputed to variability of the dimensions of the disc-shaped cells (which we shall refer to as heterogeneity) or result from what we shall term heterochrony—i.e., differences in the timing of the formation of disc-shaped cells of similar dimensions.

However, with a fitted value $f_{s}^{0}\approx 2.2$ of the meridional stretch in the anterior fold at the stage of Fig 4C, a noise level of 60% implies that at the stage in question, in some embryos, the cells in the anterior fold have not stretched at all in the meridional direction. This is consistent with the shape variability arising from differences in timing but not with the other possibility, since we previously showed that invagination in the absence of meridional stretching would lead to very flattened embryo shapes [43] unlike those observed at slightly later stages (Fig 4E). With a reduced variability of the meridional stretch in the anterior fold, the magnitude of the observed peak is unlikely to be reproduced: with a noise level of 40% in the anterior fold, compared to 6.5% for the remaining parameters, the observed peak shape variation already lies in the 99th percentile of a sample distribution of 1,000 samples of *N* = 22 perturbations each (S1 Data). We are therefore led to reject the possibility of heterogeneity of stretching. Importantly, the same qualitative shape variation arises (with a maximum in the anterior fold) if we average the shapes without allowing for local stretching of shapes (S3 Fig). This shows that this structure and hence our conclusion are robust to small perturbations of the global time stretching of the alignment.

The increased variability observed in the anterior fold thus indicates that invagination and initiation of the expansion of the anterior hemisphere (via the formation of disc-shaped cells) are really two separate, heterochronic processes. The mechanisms driving the cell shape changes in *Volvox* inversion remain unclear. Nonetheless, the observation of heterochrony sheds some light on the coupling of the two processes under discussion: if there were the same (mechanical or chemical) coupling within and between the processes, we would expect a characteristic time span and hence not expect the coupling between the processes to be noisier than the coupling within the processes. However, this cannot exclude different or additional signals: for example, one interesting possibility would be that the coupling within the invagination process is chemical, for example, but that a different signal—mechanical, for example—resulting from the invagination initiates the expansion of the anterior hemisphere. In either case, the two processes are regulated separately.

These considerations also rationalise our second observation concerning the spatial structure of shape variations, that the variation in the anterior fold is reduced as inversion of the posterior hemisphere ends (Fig 4H): there are no longer two separate processes at work. It is also interesting to note that, despite this increased variability of the meridional stretch in the anterior fold, the shape variation—both in the experimental data and in computations—has minima immediately next to the anterior fold (Fig 11B), suggesting that the shapes of these regions are robust to these particular perturbations of the intrinsic parameters. This mechanical effect underlies the observation (discussed at the end of ‘The average inversion and its local variability’) that the shape variation is reduced in the active bend region (where the cell shape changes to wedge-shaped cells driving invagination are taking place). Hence, a reduced variability of the wedge-shaped cells is not necessary, and inference of this is indeed mere teleology.

#### Peeling of the anterior hemisphere is driven by contraction

As mentioned in the introduction, type-A inversion is driven by a single wave of uniform cell shape changes travelling from the phialopore to the anterior pole; all cells successively become wedge-shaped, resulting in bending of the cell sheet [45, 54]. Similarly, invagination of the posterior hemisphere is mainly driven by cells near the equator (Fig 1D, Fig 2A), which become wedge-shaped [44], thus splaying the cells and hence imparting intrinsic curvature to the cell sheet [43, 56]. Yet no such cell wedging has been reported at the anterior fold at later stages of type-B inversion, when the anterior hemisphere peels back over the partly inverted posterior (Fig 1F, Fig 2D). Instead, the disc-shaped cells in the anterior hemisphere adopt a thin pencil-shape, and their cytoplasmic bridges move to the basal cell poles, rearranging the cells from overlapping flat discs to closely arrayed thin cells (Fig 1F, Fig 2D). This observation led to the hypothesis that these changes in cell shape and arrangement result in a decrease in surface area, which then pulls the cells over the inflection point in the anterior fold [44]. Indeed, the overall surface area of the cell sheet decreases during the peeling of the anterior hemisphere (Fig A3b, Fig A4b in S1 Text).

**Fig 12:**
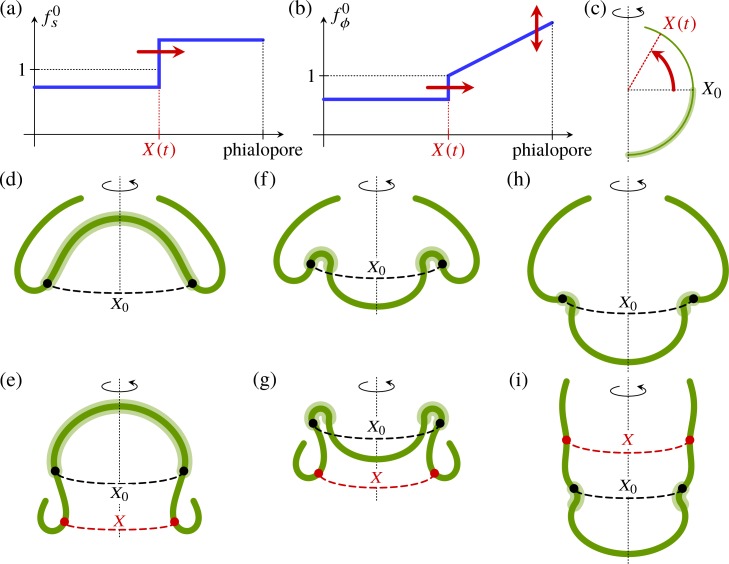
Mechanics of anterior peeling. Functional form of (a) the meridional intrinsic stretch $f_{s}^{0}$ and (b) the circumferential intrinsic stretch $f_{\phi }^{0}$ for numerical experiments. The position of the peeling front is *X*(*t*). (c) Definition of the position *X*(*t*) of the peeling front and its initial value *X*_0_ at the equator of the undeformed shell. The shaded area indicates the posterior hemisphere in which the intrinsic curvatures have opposite signs to those of the undeformed sphere. (d) Shape before peeling, with inverted posterior hemisphere. (e) Resulting shape after anterior peeling, just before phialopore closure, with *X*_0_ and *X* indicated. (f) Earlier inversion stage, where the posterior hemisphere has not yet inverted. (g) Resulting shape after peeling starting at the earlier inversion stage. (h) Even earlier stage of inversion, where the bend region has not yet been established completely. (i) Resulting shape after peeling starting at this stage, illustrating that the peeling mechanism fails if the contraction wave starts too early.

To confirm that anterior peeling can be achieved by contraction of the cell sheet alone, we perform simple quasi-static numerical experiments: we impose functional forms for the intrinsic stretches of the shell (Fig 12A–12C) representing this contraction, but we do not modify the intrinsic curvatures in the anterior hemisphere (Fig 12C). In particular, the linear variation of the circumferential stretch in the anterior hemisphere represents the different orientations of the ellipsoidal cells at the phialopore [44], where the long axis is the circumferential axis, and at the anterior fold, where the long axis is the meridional axis. To begin with, we approximate the shape in Fig 4H by a configuration with inverted posterior hemisphere (Fig 12D) and displace the intrinsic 'peeling front' (Fig 12A and 12B). The shell responds by peeling (Fig 12E), with shapes in qualitative agreement with the experimentally observed shapes. Since the peeling front is located at the anterior fold, where the shape variation is reduced during anterior peeling (Fig 4I), we again see a correlation between reduced shape variations and the location of the active cell shape changes driving inversion. This mechanism does not require posterior inversion to have completed before the contraction wave starts (Fig 12F and 12G). It is only if the contraction wave starts before the bend region has been established properly that the anterior fails to peel (Fig 12H and 12I). This shows that, provided that the contraction wave starts at a late enough stage of inversion, the two processes of posterior and anterior inversion can proceed independently, and inversion will complete after the two individual processes have completed.

These considerations suggest that contraction is sufficient to drive the peeling stage of inversion, even without changes in intrinsic curvature. This finding contradicts Kelland's [63] suggestion of a second wave of cell wedging from the equator to the phialopore as the cause of anterior peeling. Although the position of the cytoplasmic bridges (Fig 2D) on the inside end of the cells at the end of inversion [44] suggests that the intrinsic curvature may change sign in the anterior hemisphere too, this appears to be a secondary effect. Hence, intrinsic bending complements intrinsic stretching. By contrast, our previous work [43] revealed that stretching complements bending during invagination. The roles of stretching and bending are thus interchanged during inversion of the posterior and anterior hemispheres. The embryo uses these two different deformation modes for different tasks during inversion. This mirrors, at a mechanical level, the two separate processes associated to inversion of the posterior and expansion of the anterior in the discussion in ‘Expansion of the anterior hemisphere is temporally uncoupled from posterior inversion’.

## Discussion

Emerging techniques like light sheet microscopy enable an increasingly detailed observation of living, developing tissues. This leads to an ever-growing need for new approaches towards interpreting this new wealth of data. Mathematical modelling is becoming more and more important in elucidating the intricate interplay of biomechanical morphogenetic events. Accordingly, in order to develop tools that combine experimental and theoretical methods, we need biological model systems that are amenable to theoretical modelling. Owing to its simplicity, morphogenesis in *Volvox* not only reveals new mechanisms that can drive different morphogenetic processes; it also enables exploration of new approaches that are relevant to a wide variety of developmental questions.

**Fig 13:**
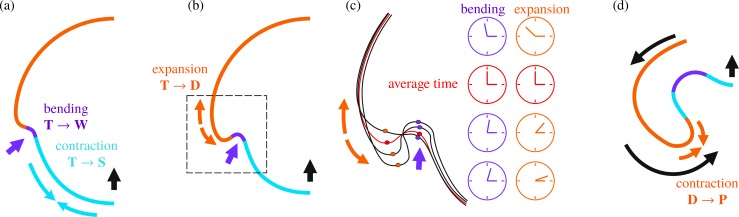
Cell sheet deformations during inversion and their relative timing. (a) Equatorial invagination is driven by cell wedging that imposes bending (purple arrows in panels a–c), while the posterior hemisphere contracts simultaneously (blue arrows). These combined changes move the posterior towards the anterior pole (black arrows in panels a–c). (b) Expansion of the anterior hemisphere is initiated in the anterior fold (orange arrows in panels b and c). (c) Detail as indicated in panel b. Illustration of the relative timing of local bending and expansion. Clock diagrams correspond to locations indicated on the shapes and represent the local timing of bending (purple) and expansion (orange) relative to the average shape (red line) and average time (red clock diagrams). There is a higher variability in the timing of expansion in the anterior fold compared to the invagination of the bend region. (d) Contraction on the inner side of the anterior fold (orange arrows) can pull cells over the inflection point and drive peeling of the anterior hemisphere (curved black arrows). Cell shape changes (**A** → **B**) are indicated in panels a,b,d as in Fig 2 and Fig 6B and 6C.

In this paper, we have combined experiment and theory to analyse the variability of *Volvox* inversion and obtain a detailed mechanical description of this process. From observations of the structure of the variability of the shapes of inverting *Volvox* embryos, we showed, using our mathematical model, that this structure requires differential regulation. Even though some discrepancies between the experimental observations and the theory remain, the simplest scenario with which the observed shape variations are consistent is that type-B inversion in *V*. *globator* results from two separately regulated processes (Fig 13A–13C), with most of the variability at the invagination stage attributed to the relative timing of these processes in individual embryos (Fig 13B and 13C). The difference between these processes is mirrored, at a mechanical level, by the different types of deformations of either hemisphere: posterior inversion mainly relies on active bending, whereas expansion and peeling of the anterior hemisphere are mainly driven by active expansion and contraction. These ideas and methods provide proof of principle of analysing morphogenesis based on its variability, and we anticipate that they can be applied to other morphogenetic events in other model organisms to add to our understanding of the regulation of morphogenesis: what kind of regulation, be it spatial or temporal, of the cell-level processes is there? This begs a further question: how does the actual amount of regulation relate to the amount required mechanically for the processes to be able to complete? Houchmandzadeh and colleagues [64] showed that diffusion of two morphogens with inhibition à la Turing [65] has error-correcting properties that can explain the precise domain specification that is observed in *Drosophila* embryos in spite of the huge variability of morphogen gradients [66]. Does the interplay of different mechanical processes yield analogous error-correcting properties?

Type-B inversion involves equatorial invagination, posterior contraction, anterior expansion, involution, and peeling of an initially spherical cell sheet (Fig 13). Numerous morphogenetic events in metazoans share these global deformations. Invagination in amphibian, echinoderm, and nematode gastrulation; in vertebrate neurulation; and *Drosophila* mesoderm formation involves the formation of bottle cells [67] that resemble the wedge-shaped cells driving invagination during inversion in *Volvox* [44]. Here, we have shown that the value of the intrinsic curvature imposed by the wedge-shaped cells remains constant, while the actual curvature increases during the initial invagination (Fig 8A). This means that while invagination is driven by active cell wedging, to which degree the cell sheet actually bends is strongly influenced by its geometry. The subsequent movement of the posterior into the anterior hemisphere is likely to be driven by a uniformly expanding wave of cell wedging moving from the equator to the posterior pole.

It has previously been noted that active contraction of the posterior hemisphere is required to allow it to move into the anterior hemisphere [43]. While we have shown here that the expansion of the anterior hemisphere is not likely to be a passive result of invagination, it remains unclear whether this expansion complements the contraction of the posterior hemisphere or whether it helps to facilitate involution of anterior cells before the phialopore begins to widen. Involution in *Xenopus* is initiated by rotation movements of cells in the central vegetal endoderm. However, in both *Xenopus* and zebrafish, cells are then pushed over the inflection point by convergent expansion of the adjacent outer cell layer [67]. During convergent expansion, cells intercalate in the circumferential direction, thereby expanding the tissue in the meridional direction. Here, we show that in *Volvox*, an alternative mechanism is sufficient to drive peeling of the anterior hemisphere: cells are being pulled over the inflection point by contraction on the inner side of the anterior fold (Fig 12, Fig 13D). This contraction of the cell sheet is achieved by a cell shape change and repositioning from disc-shaped shingled cells to pencil-shaped cells arrayed side by side (Fig 2D) [44].

### Challenges and open questions

Our answers to the questions of developmental regulation that we have raised here in the context of *Volvox* inversion have so far been either negative (i.e., ruling out certain mechanisms of regulation) or of what one might term the Occam's razor variety (i.e., invoking the law of parsimony to find the simplest modification of the model that can explain the observations). This approach of testing falsifiable hypotheses [68] has the advantage of mitigating the risk of drawing teleological conclusions. However, a fuller answer to the questions above requires estimation of the variability of all the model parameters from the experimental data. Solving this full inverse problem would provide a firmer grip on the relatively large number of fitting parameters required to reproduce the experimental observations, yet that endeavour entails significant statistical, computational, and experimental difficulties: to quantify the full range of variability, one would need a very much larger number of experimental samples to estimate the experimental distribution; additionally, for each step of the optimisation algorithm used to estimate the large number of variability parameters, a large number of computational samples would have to be computed to estimate the distribution under the model. Similar difficulties arise when estimating the variability allowed mechanically. While we have previously noted [56] that the dynamic data for type-B inversion suggest that invagination proceeds without a 'snap-through' bifurcation, there is no general requirement for individual developmental paths to lie on one and the same side of a mechanical bifurcation boundary. This poses an additional challenge for modelling approaches.

Coupling the deformations described by the elastic model to the signalling processes that underlie its intrinsic deformations is a further challenge for these models. Feedback loops relating the diffusion of contractility-inducing 'mechanogens' that are degraded by the resulting strain have been studied theoretically [69] and were coupled to a differential-tension model of a discrete epithelium [70] in more recent work [71].

After this discussion of general challenges for a mechanobiological analysis of morphogenesis and its regulation, we mention some of the remaining questions specific to *Volvox* inversion: it remains unclear what triggers the initial cell shape changes, what determines their location, and what kind of signal drives the propagation of waves of cell shape changes. It seems likely that the cytoplasmic bridges play a role in chemical or mechanical signal transduction. It is curious that inversion starts at the equator in type-B inversion but starts at the phialopore in type-A inversion. It is not known whether there are patterning mechanisms in *Volvox* that predetermine the spatial distribution of specific cell shape changes. It is unlikely that morphogens known from animals are conserved in *Volvox*, but plant hormones have been suggested to act as morphogens in photosynthetic organisms [72]. Alternatively, the position of the bend region could be determined by mechanical and/or chemical cues right at the start of inversion. Interestingly, inversion is preceded by temporary local dents in both embryo hemispheres [44]. One could speculate that this 'denting' plays a role in determining the location of the bend region. Once triggered, a wave of cell shape changes could be propagated either by mechano-sensing and/or a chemical signal. Calcium waves, for example, are known to play a role in plant development and can be triggered by mechanical stimuli [73]. In *Chlamydomonas reinhardtii*, a close relative of *Volvox*, calcium signalling plays a role, for instance, in the flagellar response [74]. Moreover, in the type-A inverter *V*. *carteri*, cells mechanically released from preinversion embryos undergo shape changes prematurely [38, 45], which could either indicate the absence of a chemical repressor [31] or that the act of isolating the cells serves itself as a mechanical cue. Combined molecular and physical approaches will be needed to address these questions. Moreover, our model does not resolve the details of the phialopore and hence does not describe the closure of the phialopore at the end of inversion, which remains a combined challenge for experiment and theory: as discussed above, the cytoplasmic bridges elongate drastically at the phialopore [44], and confocal imaging has revealed the possibility of rearrangements within the cell sheet at the phialopore. Do some cytoplasmic bridges rend to make such rearrangements possible, or are some cells next to phialopore not connected to all of their neighbour, as in type-A inversion? Understanding the details of the opening of the phialopore may also require answering more fundamental questions, the answer to which has remained elusive [38, 54]: what subcellular structures apart from endoplasmic reticulum [75] are located within the cytoplasmic bridges? How is it possible for them to stretch to such an extent? At the theoretical level, rearrangements of cells near the phialopore raise more fundamental questions of morphoelasticity [57]: in particular, how does one describe the evolution of the boundary of the manifold underlying the elastic description? Cytoplasmic bridges rending next to the phialopore would lead to the formation of lips similar to those seen in type-A inversion [45, 46]. Is there a simple theory to describe the elasticity of this nonaxisymmetric setup? We note in passing that the curling of the membranes of red blood cells upon malaria parasite egress [76] leads to shape changes qualitatively similar to the curling of the lips during type-A inversion (albeit at very different scales). These shape changes have been described theoretically by intrinsic membrane curvature [77, 78].

### Evolution of cell sheet folding in the Volvocaceae

At the close of this discussion, it is meet to briefly dwell on questions of more evolutionary flavour: all genera of Volvocaceae and its sister group Goniaceae—with the exception of the single genus *Astrephomene* [79]—display some form of inversion [42]. There is a general trend among these genera for complexity of inversion to increase with cell number, enabling comparative studies of the evolution of this complexity [49]. The simplest inversion occurs in *Gonium* [48]: as cells uniformly change their shape, the initially bowl-shaped, convex embryos become concave. Increases of this complexity may appear in different guises: certain cell shape changes may arise only in part of the cell sheet, as in *Pleodorina* [49], or cell shape changes may proceed in a wave, as exemplified by type-A inversion in *Volvox* [45]. The separate regulation of different processes and heterochrony in type-B inversion described here may be a prototype of an additional trait of the evolution of multicellularity that can be studied in the volvocine algae: the transition between cell sheet deformations driven by a single process and those resulting from two separate processes. This complements the similarly prototypical transition from organisms with one cell type to organisms with two cell types associated with germ-soma differentiation in the volvocine algae [80]. The question how the different species of the polyphyletic genus *Volvox* [81] evolved different ways of turning themselves inside out remains, however. Phylogenetic studies of the volvocine algae show that different inversion types evolved several times independently in different lineages [46, 82]. Additionally, Pocock [83] reported that in *V*. *rousseletii* and *V*. *capensis*, inversion type depends on the (sexual or asexual) reproduction mode. This may be a manifestation of the poorly understood role of environmental and evolutionary cues in morphogenesis [84], but such cues remain subject to the mechanical constraints on the respective tissue.

## Materials and methods

### Acquisition of experimental data

Wild-type strain *V*. *globator* Linné (SAG 199.80) was obtained from the Culture Collection of Algae at the University of Göttingen, Germany [85], and cultured as previously described [86] with a cycle of 16 h light at 24°C and 8 h dark at 22°C.

#### SPIM

**Fig 14:**
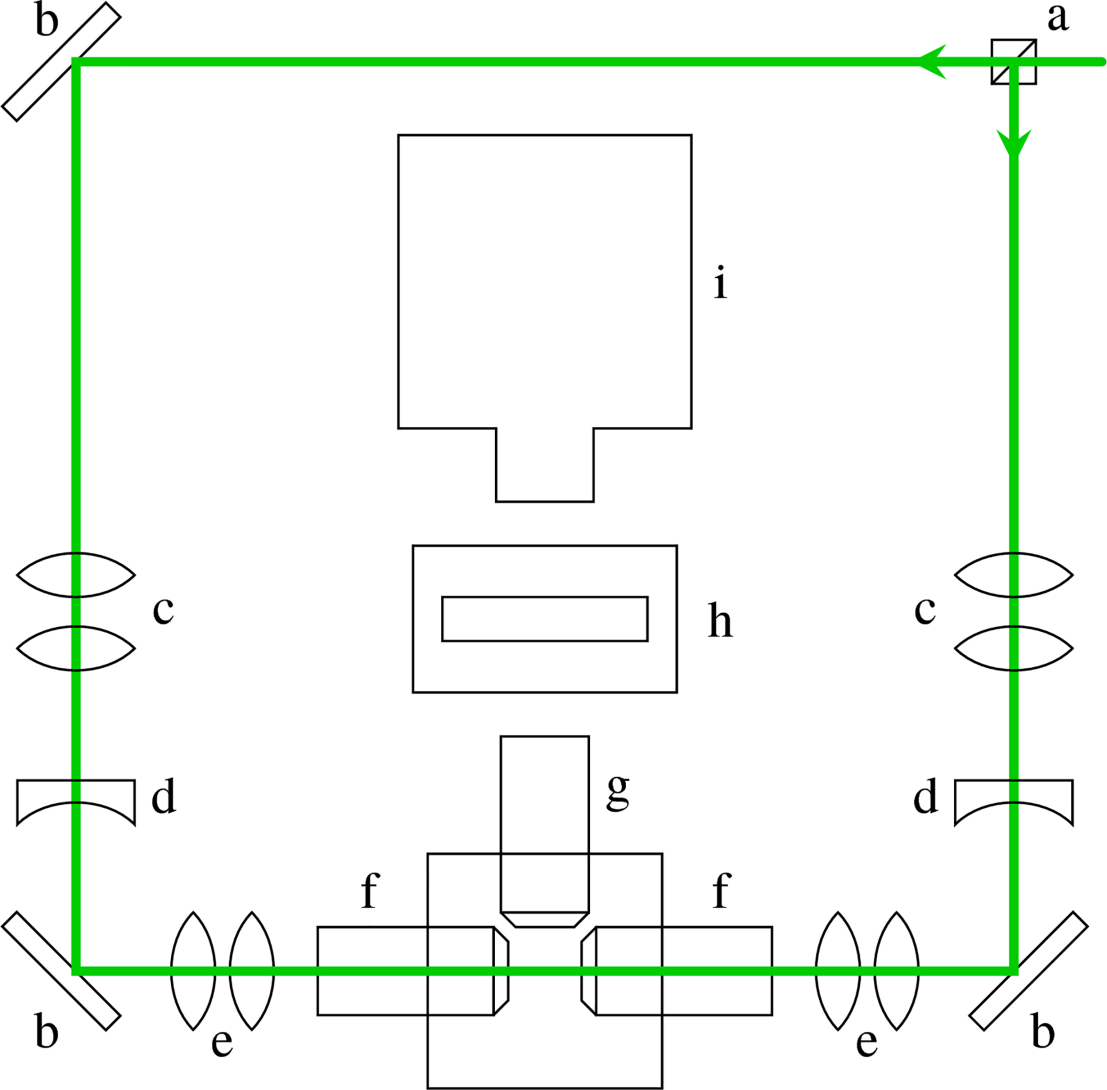
SPIM imaging setup. a: beamsplitter cube, b: mirror, c: beam expander, d: cylindrical lens, e: telescope, f: illumination objective, g: detection objective, h: emission filter, i: camera. SPIM, selective plane illumination microscopy.

A selective plane illumination microscopy system was assembled based on the OpenSPIM setup [60], with modifications to accommodate a Stradus Versalase laser system (Vortran Laser Technology, Sacramento, CA, United States of America) and a CoolSnap Myo CCD camera (1,940 × 1,460 pixels; Photometrics, AZ, USA). Moreover, to decrease the loss of data due to shadowing, a second illumination arm was added to the setup (Fig 14). Illumination from both sides improved the image quality and enabled reslicing of the z-stacks when embryos began to spin during anterior inversion.

*V*. *globator* parent spheroids were mounted in a column of low-melting-point agarose and suspended in fluid medium in the sample chamber. To visualise the cell sheet deformations of inverting *V*. *globator* embryos, chlorophyll autofluorescence was excited at λ = 561 nm and detected at λ > 570 nm. Z-stacks were recorded at intervals of 60 s over 4–6 h to capture inversion of all embryos in a parent spheroid. We acquired time-lapse data of 13 different parent spheroids, each containing 4–7 embryos.

#### Confocal laser scanning microscopy

Samples were immobilised on glass-bottom dishes by embedding them in low-melting-point agarose and then covered with fluid medium. Chlorophyll autofluorescence was excited at λ = 639 nm and detected at λ > 647 nm. Z-stacks were recorded at intervals of 30 s over 1–2 h to capture inversion of a single embryo. Trajectories of individual cells close to the phialopore were obtained using Fiji [87]. Experiments were carried out using an Observer Z1 spinning disk microscope (Zeiss, Germany).

#### Image tracing

To ensure optimal image quality (traceability) for the quantitative analyses of inversion, from the inversion processes recorded with SPIM, we selected 11 inversions (in 6 different parent spheroids) in which the acquisition plane was initially approximately parallel to the midsagittal plane of the embryos. Midsagittal cross-sections were obtained using Fiji [87] and Amira (FEI, OR, USA).

Splines were fitted to these cross-sections using the following semiautomated approach implemented in Python/C++ (S1 Code): in a preprocessing step, images were bandpass filtered to remove short-range noise and large-range intensity correlations. Low-variance gaussian filters were applied to smooth out the images slightly. Splines were obtained from the preprocessed images *I*(***x***) using the active contour model [88], with modifications to deal with intensity variations and noise in the image: the spline ***x***_s_(*s*), where *s* is arclength, minimises an energy

$$E[x_{s}]=E_{\mathrm{image}}[x_{s}]+E_{\mathrm{spline}}[x_{s}]+E_{\mathrm{skel}}[x_{s}],$$ (1)

where

$$E_{\mathrm{image}}[x_{s}]=-\alpha \int I\left(x_{s}(s)\right)ds,$$ (2)

$$E_{\mathrm{spline}}[x_{s}]=\beta \int {\left\|\frac{\partial^{2}x_{s}}{\partial s^{2}}\right\|}^{2}ds+\gamma {\left(\int ds-L_{0}\right)}^{2},$$ (3)

$$E_{\mathrm{skel}}[x_{s}]=\delta \int I_{\mathrm{skel}}\left(x_{s}(s)\right)\;ds,$$ (4)

wherein *α*,*β*,*γ*,*δ* are parameters, *L*_0_ is the estimated length of the shape outline, and *I*_skel_ is obtained by skeletonising *I* using the algorithm of [89] to minimise the number of branches.

The energy E was minimised using stochastic gradient descent. Initial guesses for the splines were obtained by manually initialising about 15 timepoints for each inversion using a few guide points and polynomial interpolation. An initial guess for other frames was obtained from these frames by interpolation; these interpolated shapes were used to estimate *L*_0_.

With *δ* = 0, the standard active contour model of [88] is recovered. We found that this model was not sufficient to yield fits of acceptable quality, because of the existence of local minima at small values of *α*, while larger values of *α* lead to noisy splines. Thresholding methods on their own were not sufficient either, because of branching and, in particular, since they failed to capture the bend region properly. Dynamic thresholding methods as in [90] are not applicable either, because of the fast variations of the brightness of the images. The modified active contour model did, however, produce good fits when we progressively reduced *δ* to 0 with increasing iteration number of the minimisation scheme, yielding smooth splines, while overcoming the local minima (or, from the point of view of the skeletonisation method, choosing the correct, branchless part of the skeleton). All outlines obtained from this algorithm were manually checked and corrected.

### Analysis of traced embryo shapes

From the traced cell sheet outlines, anterior–posterior axes of the embryos were determined as follows: for shapes for which the bend region was visible on either side of the cross-section, the embryo axis was defined to be the line through the centre of mass of the shape that is perpendicular to the common tangent to the two bend regions (the apex line). Shapes were then rotated and translated manually so that their axes coincided. Since embryos do not rotate much before the flagella grow, the orientation of the axes of the earliest traces (for which the bend regions are not apparent) were taken to be the same as that of the earliest timepoint for which two bend regions were visible. The intersection of the embryo trace and axis defines the posterior pole. After manually recentring some embryos with more pronounced asymmetry, embryos were halved to obtain *N* = 22 embryo halves.

#### Aligning and averaging embryo shapes

**Fig 15:**
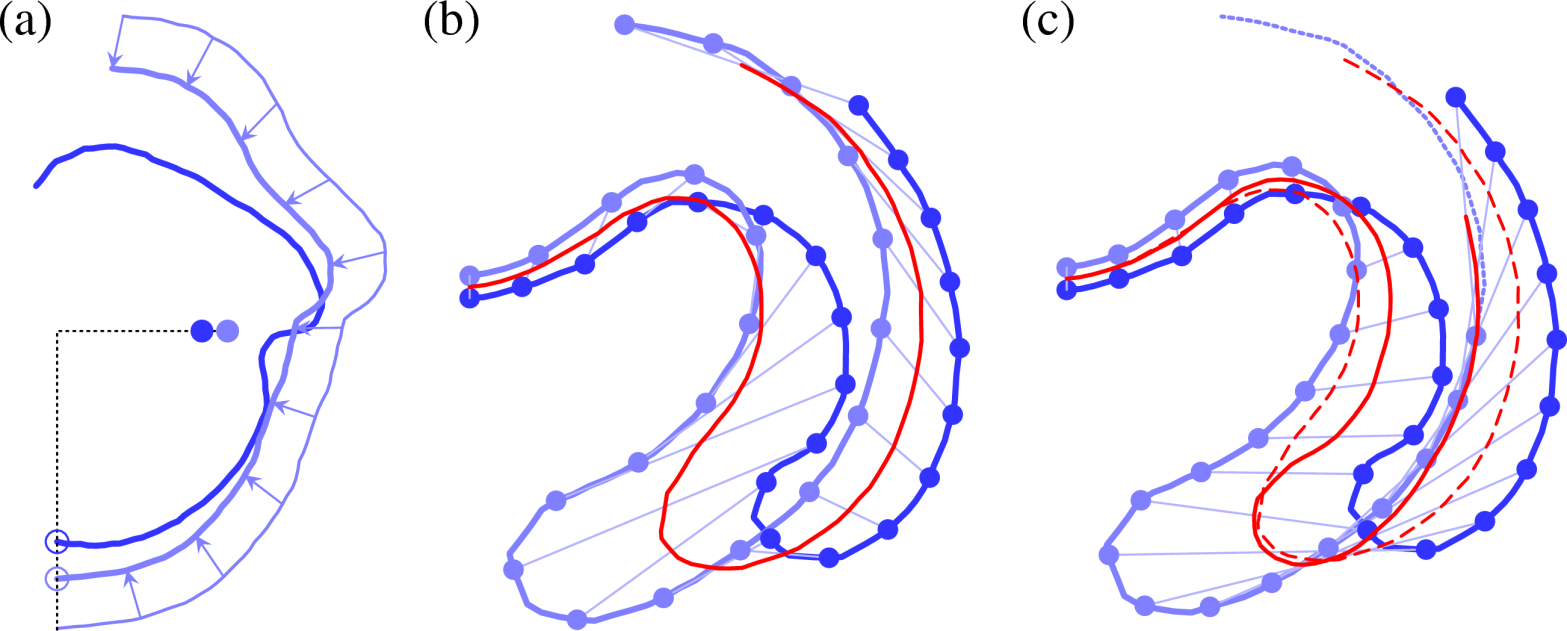
Geometry of averaging. Alignment and local dilation of embryo shapes. (a) Degrees of freedom for aligning shapes: after scaling and horizontal alignment by posterior pole (empty circles), only vertical alignment of shapes remains to be imposed by aligning centres of mass (filled circles). (b) Distributing points along arclength by averaging over different total arclengths ensures that the rims of the respective phialopores are matched up. Red line: average shape. (c) Distributing points along arclength at fixed distance between fitting points may yield a more faithful representation of part of the shape but does not match up phialopores. Solid red line: average shape; dashed red line: average from b for comparison.

To align embryos to each other, one embryo half was arbitrarily taken as the reference shape, and *T* = 10 regularly spaced timepoints were chosen for fitting. (These timepoints were chosen to be well after invagination had started and before the phialopore had closed so that defining the start and end of inversion was not required.) For each of the remaining *N*−1 embryo halves, a scale and *T* corresponding timepoints were then sought, with shapes being (linearly) interpolated at intermediate timepoints. The interpolated and scaled shapes were centred so that the centres of mass of the cross-sections coincided. This fixes the degree of freedom of translation parallel to the embryo axis; the position perpendicular to the axis is fixed by requiring that the embryo axes coincide (Fig 15A). The motivation for using the centres of mass of the cross-sections (rather than of that of the embryos, which assigns the same mass to each cell by assigning more mass to those points of the cross-section that are farther away from the embryo axis) is a biological one: because of the cylindrical symmetry of the cell shape changes, this average assigns the same mass to each cell shape change.

For aligning embryo shapes, we distribute *M* = 100 averaging points uniformly along the (possibly different) arclength of each of the embryo halves. Corresponding points were determined by dynamic warping of shapes using the dynamic ‘time’ warping algorithm described, for example, in [91], and the distances between these shapes and their averages were minimised as explained in what follows. The parameters describing the alignment are thus the scale factors *S*_1_ = 1,*S*_2_,…*S*_*N*_ and the averaging time points ***τ***_1_ = (*τ*_11_,*τ*_12_,…,*τ*_1*T*_), ***τ***_2_,…,***τ***_*N*_, where ***τ***_1_ is fixed. Each choice of these parameters yields a set of shapes ***X***_1_ = (*x*_11_,…,*x*_1*M*_),***X***_2_,…,***X***_*N*_ with points matched up by maps *σ*_1_,*σ*_2_,…,*σ*_*N*_ obtained from the dynamic warping algorithm. The effect of the local stretching allowed by this algorithm is illustrated in Fig 15B and 15C. The mean shapes having been determined, the sum of euclidean distances between shapes of individual embryos and the mean,

$$\sum_{t=1}^{T}{\left\{\sum_{n=1}^{N}\sum_{m=1}^{M}{\left(x_{n\sigma_{n}(m)}-{\bar{x}}_{m}\right)}^{2}\right\}}^{1/2},\;\mathrm{where}\;{\bar{x}}_{m}=\frac{1}{N}\sum_{n=1}^{N}x_{n\sigma_{n}(m)},$$ (5)

was minimised over the space of all these alignment parameters using the Matlab (The MathWorks) routine fminsearch, modified to incorporate the variant of the Nelder–Mead algorithm suggested in [92] for problems with a large number of parameters. After the algorithm had converged, each of the alignment parameters was modified randomly, and the algorithm was run again. This was repeated until the alignment score defined by Eq (5) did not decrease further. The means ${\bar{x}}_{1},{\bar{x}}_{2},\ldots ,{\bar{x}}_{M}$ for the alignment minimising Eq (5) define the average embryo shapes. Sample code is given in S2 Code.

Aligning shapes in this way using dynamic warping requires a considerable amount of computer time. To make the problem computationally tractable, we invoked the usual heuristics of only computing pairwise distances and reducing the size of the dynamic programming matrix by only computing a band centred on the diagonal. To verify the algorithm, we also ran several instantiations of the alignment algorithm without dynamic warping (i.e., with *σ*_*n*_ = id) and with larger parameter randomisations, confirming that the modified Nelder–Mead algorithm finds an appropriate alignment. This also enabled us to verify that results do not change qualitatively if the centres of mass of the cross-sections are replaced with those of the embryo halves (even though, as noted in the main text, the shapes without dynamic warping of shapes are unsatisfactory, since they have kinks in the bend region that are not seen in individual embryo shapes).

For the simple alternative averaging method in S2 Fig, different numbers of averaging points were distributed at equal arclength spacing along all individual shapes. Differences in arclengths of individual embryos mean that the rims of the phialopores of individual embryo halves are not necessarily matched up (Fig 15C). No time stretching was applied. The averaging method in S3 Fig is the method discussed above, without dynamic warping of shapes (i.e., with *σ*_*n*_ = id).

### Elastic model

**Fig 16:**
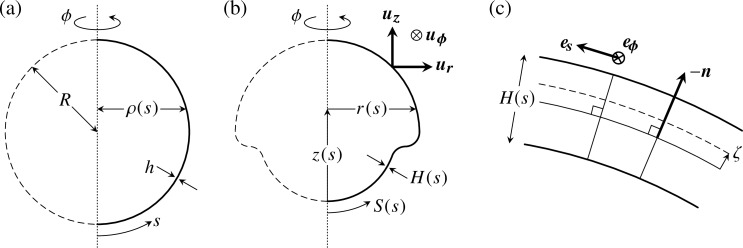
Geometry of the problem. (a) Undeformed geometry: a spherical shell of radius *R* and thickness *h* ≪ *R* is characterised by its arclength *s* and distance from the axis of revolution *ρ*(*s*). (b) Deformed configuration characterised by arclength *S*(*s*) and distance *r*(*s*) from the axis of revolution. Intrinsic volume conservation sets the thickness *H*(*s*) of the sheet. A local basis (***u***_***r***_,***u***_***ϕ***_,***u***_***z***_) describes the deformed surface. (c) Cross-section of the shell under the Kirchhoff hypothesis, with a coordinate *ζ* across the thickness of the shell, parallel to the normal ***n*** to the midsurface.

We consider a spherical shell of radius *R* and uniform thickness *h* ≪ *R* (Fig 16A), characterised by its arclength *s* and distance from the axis of revolution *ρ*(*s*), to which correspond arclength *S*(*s*) and distance from the axis of revolution *r*(*s*) in the axisymmetric deformed configuration (Fig 16B). We define the meridional and circumferential stretches

$$f_{s}(s)=\frac{dS}{ds},\;f_{\phi }(s)=\frac{r(s)}{\rho (s)}.$$ (6)

The position vector of a point on the midsurface of the deformed shell is thus

$$r(s,\phi )=r(s)u_{r}(\phi )+z(s)u_{z},$$ (7)

in a right-handed set of axes (***u***_***r***_,***u***_***ϕ***_,***u***_***z***_), and so the tangent vectors to the deformed midsurface are

$$e_{s}=r^{\prime }u_{r}+z^{\prime }u_{z},e_{\phi }=ru_{\phi },$$ (8)

where dashes denote differentiation with respect to *s*. By definition, ${r^{\prime }}^{2}+{z^{\prime }}^{2}=f_{s}^{2}$, and so we may write

$$r^{\prime }=f_{s}\cos\beta ,\;z^{\prime }=f_{s}\sin\beta .$$ (9)

Hence, the normal to the deformed midsurface is

$$n=\frac{r^{\prime }u_{z}-z^{\prime }u_{r}}{f_{s}}=\cos\beta \;u_{z}-\sin\beta \;u_{r}.$$ (10)

We now make the Kirchhoff 'hypothesis' [58] that the normals to the undeformed midsurface remain normal to the deformed midsurface (Fig 16C). Taking a coordinate *ζ* across the thickness *h* of the undeformed shell, the position vector of a general point in the shell is

$$r(s,\phi ,\zeta )=ru_{r}+zu_{z}+\zeta n=(r-\zeta \sin\beta )u_{r}+(z+\zeta \cos\beta )u_{z}.$$ (11)

The tangent vectors to the shell are thus

$$e_{s}=f_{s}(1-\kappa_{s}\zeta )(\cos\beta \;u_{r}+\sin\beta \;u_{z}),\;e_{\phi }=\rho f_{\phi }(1-\kappa_{\phi }\zeta )u_{\phi },$$ (12)

where *κ*_*s*_ = *β*′/*f*_*s*_ and *κ*_*ϕ*_ = sin *β*/*r* are the curvatures of the deformed midsurface. The metric of the deformed shell under the Kirchhoff hypothesis accordingly takes the form

$$dr^{2}=f_{s}^{2}{\left(1-\kappa_{s}\zeta \right)}^{2}ds^{2}+f_{\phi }^{2}{\left(1-\kappa_{\phi }\zeta \right)}^{2}\rho^{2}d\phi^{2}.$$ (13)

The geometric and intrinsic deformation gradient tensors are thus

$$F^{g}=\left(\begin{array}{cc} f_{s}(1-\kappa_{s}\zeta ) & 0\\ 0 & f_{\phi }(1-\kappa_{\phi }\zeta ) \end{array}\right),\;F^{0}=\left(\begin{array}{cc} f_{s}^{0}(1-\kappa_{s}^{0}\zeta ) & 0\\ 0 & f_{\phi }^{0}(1-\kappa_{\phi }^{0}\zeta ) \end{array}\right),$$ (14)

where $f_{s}^{0},f_{\phi }^{0}$ and $\kappa_{s}^{0},\kappa_{\phi }^{0}$ are the intrinsic stretches and curvatures of the shell. Thence, invoking the standard multiplicative decomposition of morphoelasticity [57], the elastic deformation gradient tensor is

$$F=F^{g}{\left(F^{0}\right)}^{-1}=\left(\begin{array}{cc} \frac{f_{s}(1-\kappa_{s}\zeta )}{f_{s}^{0}(1-\kappa_{s}^{0}\zeta )} & 0\\ 0 & \frac{f_{\phi }(1-\kappa_{\phi }\zeta )}{f_{\phi }^{0}(1-\kappa_{\phi }^{0}\zeta )} \end{array}\right).$$ (15)

While we do not make any assumption about the geometric or intrinsic strains derived from **F**^**g**^ and **F**^**0**^, respectively, we assume that the elastic strains derived from **F** remain small; we may thus approximate

$$\varepsilon_{ss}\approx \frac{f_{s}(1-\kappa_{s}\zeta )}{f_{s}^{0}(1-\kappa_{s}^{0}\zeta )}-1,\;\varepsilon_{\phi \phi }\approx \frac{f_{s}(1-\kappa_{s}\zeta )}{f_{s}^{0}(1-\kappa_{s}^{0}\zeta )}-1,$$ (16)

with the off-diagonal elements vanishing, *ε*_*sϕ*_ = *ε*_*ϕs*_ = 0. For a hookean material with elastic modulus *E* and Poisson's ratio *ν* [58, 59], the elastic energy density (per unit extent in the meridional direction) is found by integrating across the thickness of the shell:

$$\begin{array}{l} \frac{E}{2\pi \rho }=\frac{E}{2(1-\nu^{2})}\int_{-h/2}^{h/2}\left(\varepsilon_{ss}^{2}+\varepsilon_{\phi \phi }^{2}+2\nu \varepsilon_{ss}\varepsilon_{\phi \phi }\right)d\zeta \\ \;=\frac{Eh}{2(1-\nu^{2})}\{\left(1+\frac{h^{2}}{4}{\left(\kappa_{s}^{0}\right)}^{2}\right)E_{s}^{2}+\left(1+\frac{h^{2}}{4}{\left(\kappa_{\phi }^{0}\right)}^{2}\right)E_{\phi }^{2}\\ \;+2\nu \left(1+\frac{h^{2}}{12}\left({\left(\kappa_{s}^{0}\right)}^{2}+\kappa_{s}^{0}\kappa_{\phi }^{0}+{\left(\kappa_{\phi }^{0}\right)}^{2}\right)\right)E_{s}E_{\phi }\}\\ \;+\frac{Eh^{3}}{24(1-\nu^{2})}\{K_{s}^{2}+K_{\phi }^{2}+2\nu K_{s}K_{\phi }-4\kappa_{s}^{0}E_{s}K_{s}-4\kappa_{\phi }^{0}E_{\phi }K_{\phi }\\ \;-2\nu \left(\kappa_{s}^{0}+\kappa_{\phi }^{0}\right)\left(E_{\phi }K_{s}+E_{s}K_{\phi }\right)\}, \end{array}$$ (17)

where we have expanded the energy up to third order in the thickness and where we have defined the shell strains and curvature strains

$$E_{s}=\frac{f_{s}-f_{s}^{0}}{f_{s}^{0}},\;E_{\phi }=\frac{f_{\phi }-f_{\phi }^{0}}{f_{\phi }^{0}},\;K_{s}=\frac{f_{s}\kappa_{s}-f_{s}^{0}\kappa_{s}^{0}}{f_{s}^{0}},\;K_{\phi }=\frac{f_{\phi }\kappa_{\phi }-f_{\phi }^{0}\kappa_{\phi }^{0}}{f_{\phi }^{0}}.$$ (18)

As in our previous work [43, 56], the elastic modulus is an overall constant that ensures that E has units of energy but does not otherwise affect the shapes. This property of the model enables us to neglect global variations of the elastic modulus between different embryos. We make the additional assumption that the elastic modulus does not vary locally within embryos. We shall also assume that *ν* = 1/2 for incompressible biological material; the cell size measurements of [62] for type-A inversion in *V*. *carteri* support this assumption qualitatively. (These considerations also explain why we do not perturb these mechanical parameters in our analysis of the shape variations.) We finally set *h*/*R* = 0.15 as in our previous work.

#### Derivation of the governing equations

The derivation of the governing equations proceeds similarly to standard shell theories [58, 59, 93]. In fact, the resulting equations turn out to have a form very similar to those of standard shell theories, but a host of extra terms arise in the expressions for the shell stresses and moments because of the assumptions of morphoelasticity. The variation of the elastic energy takes the form

$$\frac{\delta E}{2\pi \rho }=n_{s}\delta E_{s}+n_{\phi }\delta E_{\phi }+m_{s}\delta K_{\phi }+m_{\phi }\delta K_{\phi },$$ (19)

with

$$\delta E_{s}=\frac{\delta f_{s}}{f_{s}^{0}}=\frac{1}{f_{s}^{0}}(\sec\beta \;\delta r^{\prime }+f_{s}\tan\beta \;\delta \beta ),\;\delta E_{\phi }=\frac{\delta f_{\phi }}{f_{\phi }^{0}}=\frac{\delta r}{f_{\phi }^{0}\rho },$$ (20)

$$\delta K_{s}=\frac{\delta (f_{s}\kappa_{s})}{f_{s}^{0}}=\frac{\delta \beta^{\prime }}{f_{s}^{0}},\;\delta K_{\phi }=\frac{\delta (f_{\phi }\kappa_{\phi })}{f_{\phi }^{0}}=\frac{\cos\beta }{f_{\phi }^{0}\rho }\delta \beta ,$$ (21)

wherein dashes again denote differentiation with respect to *s* and wherein the shell stresses and moments are defined by

$$\begin{array}{l} n_{s}=\frac{Eh}{1-\nu^{2}}\{E_{s}+\nu E_{\phi }+\frac{h^{2}}{12}(3{\left(\kappa_{s}^{0}\right)}^{2}E_{s}+\nu \left({\left(\kappa_{s}^{0}\right)}^{2}+\kappa_{s}^{0}\kappa_{\phi }^{0}+{\left(\kappa_{\phi }^{0}\right)}^{2}\right)E_{\phi }\\ \;-2\kappa_{s}^{0}K_{s}-\nu \left(\kappa_{s}^{0}+\kappa_{\phi }^{0}\right)K_{\phi })\}, \end{array}$$ (22)

$$\begin{array}{l} n_{\phi }=\frac{Eh}{1-\nu^{2}}\{E_{\phi }+\nu E_{s}+\frac{h^{2}}{12}(3{\left(\kappa_{\phi }^{0}\right)}^{2}E_{\phi }+\nu \left({\left(\kappa_{s}^{0}\right)}^{2}+\kappa_{s}^{0}\kappa_{\phi }^{0}+{\left(\kappa_{\phi }^{0}\right)}^{2}\right)E_{s}\\ \;-2\kappa_{\phi }^{0}K_{\phi }-\nu \left(\kappa_{s}^{0}+\kappa_{\phi }^{0}\right)K_{s})\}, \end{array}$$ (23)

and

$$m_{s}=\frac{Eh^{3}}{12(1-\nu^{2})}\left\{K_{s}+\nu K_{\phi }-2\kappa_{s}^{0}E_{s}-\nu \left(\kappa_{s}^{0}+\kappa_{\phi }^{0}\right)E_{\phi }\right\},$$ (24)

$$m_{\phi }=\frac{Eh^{3}}{12(1-\nu^{2})}\left\{K_{\phi }+\nu K_{s}-2\kappa_{\phi }^{0}E_{\phi }-\nu \left(\kappa_{s}^{0}+\kappa_{\phi }^{0}\right)E_{s}\right\}.$$ (25)

Defining

$$N_{s}=\frac{n_{s}}{f_{s}^{0}f_{\phi }},\;N_{\phi }=\frac{n_{\phi }}{f_{\phi }^{0}f_{s}},\;M_{s}=\frac{m_{s}}{f_{s}^{0}f_{\phi }},\;M_{\phi }=\frac{m_{\phi }}{f_{\phi }^{0}f_{s}},$$ (26)

the variation becomes

$$\begin{array}{l} \frac{\delta E}{2\pi }=rN_{s}\sec\beta \;\delta r+rM_{s}\;\delta \beta -\int \left(\frac{d}{ds}\left(rN_{s}\sec\beta \right)-f_{s}N_{\phi }\right)\delta r\;ds\\ \;+\int \left(rf_{s}N_{s}\tan\beta +f_{s}M_{\phi }\cos\beta -\frac{d}{ds}\left(rM_{s}\right)\right)\delta \beta \;ds. \end{array}$$ (27)

The Euler–Lagrange equations of Eq (17) are thus

$$\frac{d}{ds}\left(rN_{s}\sec\beta \right)-f_{s}N_{\phi }=0,\;\frac{d}{ds}\left(rM_{s}\right)-f_{s}M_{\phi }\cos\beta -rf_{s}N_{s}\tan\beta =0.$$ (28)

To remove the singularity that arises in the second of Eq (28) when *β* = *π*/2, we define the transverse shear tension *T* = −*N*_*s*_ tan *β* as in standard shell theories. The governing equations can then be rearranged to give

$$\frac{dN_{s}}{ds}=f_{s}\left(\frac{N_{\phi }-N_{s}}{r}\cos\beta +\kappa_{s}T\right),\;\frac{dM_{s}}{ds}=f_{s}\left(\frac{M_{\phi }-M_{s}}{r}\cos\beta -T\right).$$ (29)

By differentiating the definition of *T* and using the first of Eq (29), one finds that

$$\frac{dT}{ds}=-f_{s}\left(\kappa_{s}N_{s}+\kappa_{\phi }N_{\phi }+\frac{T}{r}\cos\beta \right).$$ (30)

Together with the geometrical equations *r*′ = *f*_*s*_ cos *β* and *β*′ = *f*_*s*_*κ*_*s*_, Eqs (29) and (30) describe the deformed shell. The five required boundary conditions can be read off the variation Eq (27) and the definition of *T*,

β=0,r=0,T=0,attheposteriorpole, (31)

$$N_{s}=0,\;M_{s}=0,\;\mathrm{at}\;\mathrm{the}\;\mathrm{phialopore}.$$ (32)

We solve these equations numerically using the boundary value–problem solver bvp4c of Matlab (The MathWorks). At each step of the integration, *f*_*s*_ and *κ*_*s*_ are determined from the solution of the system of linear equations relating *N*_*s*_,*M*_*s*_ and *f*_*s*_,*f*_*s*_*κ*_*s*_ that is obtained by combining Eqs (22), (24), and (26). This allows computation of *N*_*ϕ*_ and *M*_*ϕ*_, continuing the integration. A Matlab implementation of the governing equations is given in S2 Code.

For completeness, we note that if external forces are applied to the shell, and δW is the variation of the work done by these forces, then the variational condition is δE+δW=0. In that case, it is useful to write the variation Eq (27) in terms of *δr* and *δz*. We note that *δr*′ = −*f*_*s*_ sin *β δβ* and *δz*′ = *f*_*s*_ cos *β δβ*, and so

$$f_{s}\;\delta \beta =\cos\beta \;\delta z^{\prime }-\sin\beta \;\delta r^{\prime }.$$ (33)

Using this geometric relation and integrating by parts, we obtain

$$\begin{array}{l} \frac{\delta E}{2\pi }=rM_{s}\;\delta \beta +\left\{rN_{s}\cos\beta -\frac{\sin\beta }{f_{s}}\left(M_{\phi }\cos\beta -\frac{d}{ds}(rM_{s})\right)\right\}\delta r\\ \;+\left\{rN_{s}\sin\beta +\frac{\cos\beta }{f_{s}}\left(M_{\phi }\cos\beta -\frac{d}{ds}(rM_{s})\right)\right\}\delta z\\ \;+\int \left\{f_{s}N_{\phi }-\frac{d}{ds}\left(rN_{s}\cos\beta -\frac{\sin\beta }{f_{s}}\left(M_{\phi }\cos\beta -\frac{d}{ds}(rM_{s})\right)\right)\;\right\}\delta r\;ds\\ \;-\int \frac{d}{ds}\left(rN_{s}\sin\beta +\frac{\cos\beta }{f_{s}}\left\{M_{\phi }\cos\beta -\frac{d}{ds}(rM_{s})\right\}\right)\delta z\;ds. \end{array}$$ (34)

#### Limitations of the theory

The theory presented here has a singularity in a biologically relevant limit: the intrinsic deformation gradient F^0^ becomes singular at $\left|\kappa_{s}^{0}\right|={\left(h/2\right)}^{-1}$ or $\left|\kappa_{\phi }^{0}\right|={\left(h/2\right)}^{-1}$. This value corresponds precisely to the case of cells that are constricted to a point at one cell pole.

A related issue that has only cropped up implicitly in the above derivation is intrinsic volume conservation: since we assume the cell sheet to be incompressible, its intrinsic deformations are accompanied by variations of its intrinsic thickness. The absence of a jacobian factor in the integration of the energy density with respect to the coordinates of the undeformed spherical shell above is the mathematical consequence of this. Intrinsic volume conservation is perhaps the main difference between the present theory for elastic shells and the theory of incompatible elastic plates of [94]. It is instructive to discuss the intrinsic thickness *H*(*s*) of the shell in some more detail. By assumption, *H* is close to the thickness of the deformed shell but differs from the thickness *h* of the undeformed shell. For a doubly curved shell, intrinsic volume conservation implies that the relative thickness *η* = *H*/*h* is a function of both the intrinsic stretches $f_{s}^{0},f_{\phi }^{0}$ and the intrinsic curvatures $\kappa_{s}^{0},\kappa_{\phi }^{0}$. In more detail, the volume of an element of shell is

$$\int_{-H/2}^{H/2}f_{s}^{0}f_{\phi }^{0}\left(1-\kappa_{s}^{0}\zeta \right)\left(1-\kappa_{\phi }^{0}\zeta \right)\rho \;ds\;d\phi \;d\zeta =f_{s}^{0}f_{\phi }^{0}H\left(1+\frac{H^{2}}{12}\kappa_{s}^{0}\kappa_{\phi }^{0}\right)\rho \;ds\;d\phi .$$ (35)

It follows that *η* satisfies the cubic equation

$$\left(\frac{h^{2}}{12}f_{s}^{0}f_{\phi }^{0}\kappa_{s}^{0}\kappa_{\phi }^{0}\right)\eta^{3}+f_{s}^{0}f_{\phi }^{0}\eta -\left(1+\frac{h^{2}}{12R^{2}}\right)=0,$$ (36)

the solution of which can be expressed in closed form. It is clear that this equation always has a solution if $\kappa_{s}^{0}\kappa_{\phi }^{0}>0$. If $\kappa_{s}^{0}\kappa_{\phi }^{0}<0$, there is a solution if and only if

$$|\kappa_{s}^{0}\kappa_{\phi }^{0}|<{\left(\frac{4f_{s}^{0}f_{\phi }^{0}}{3h}\right)}^{2}{\left(1+\frac{h^{2}}{12R^{2}}\right)}^{-2}.$$ (37)

Since 16/9 < 4, this condition may fail before the intrinsic geometry becomes singular, so this additional condition is not vacuous. This brief discussion therefore points to some interesting, more fundamental problems in the theory of morphoelastic shells.

There is an additional subtlety associated with the geometric and intrinsic deformation gradient tensors in Eq (14): the components of **F**^**g**^ are expressed in Eq (14) relative to the (natural) mixed basis $\left\{{\hat{e}}_{s},{\hat{e}}_{\phi }\right\}⊗\left\{{\hat{E}}_{s},{\hat{E}}_{\phi }\right\}$, where ${\hat{e}}_{s},{\hat{e}}_{\phi }$ are the unit vectors tangent to the deformed configuration of the shell, and ${\hat{E}}_{s},{\hat{E}}_{\phi }$ are defined analogously for the undeformed configuration. We have implicitly written down the components of **F**^**0**^ relative to the same basis. In general, however, the components of **F**^**0**^ in Eq (14) are those relative to the basis $\left\{{\hat{e}}_{s}^{0},{\hat{e}}_{\phi }^{0}\right\}⊗\left\{{\hat{E}}_{s},{\hat{E}}_{\phi }\right\}$, in which the unit basis $\left\{{\hat{e}}_{s}^{0},{\hat{e}}_{\phi }^{0}\right\}$ can a priori be specified freely. We have neglected these additional degrees of freedom in the above derivation; the question of how to define a natural intrinsic tangent basis $\left\{{\hat{e}}_{s}^{0},{\hat{e}}_{\phi }^{0}\right\}$ is, however, an interesting one, since the intrinsic stretches and curvatures need not be compatible.

### Fitting embryo shapes

For the purpose of fitting the model to the observed average shapes, we fit values of the 15 parameters *f*_1_,…,*f*_5_, *κ*_1_,…,*κ*_5_, *s*_1_,…,*s*_5_ defined in Fig 6D–6F. The other geometrical parameter of the shell, the angular extent *P* of the phialopore, is not fitted for. We arbitrarily set *P* = 0.3. The reasons for this simplification are discussed in the main text. We do not fit either for the distance Δ*s* over which we regularise the functional form of $\kappa_{\phi }^{0}$ (Fig 6F), since we lack information about the cell shape changes that define it. We arbitrarily set Δ*s* = 0.05.

Numerical shapes were fitted to the average shapes by distributing *M* = 100 points uniformly along the arclength of the numerical and average shapes and minimising a euclidean distance between them using the Matlab (The MathWorks) routine fminsearch, modified to incorporate the variant of the Nelder–Mead algorithm of [92] as well as a modified shrinking step of the Nelder–Mead simplex. A custom-written adaptive stepper was used to move about in parameter space and select the initial guess for the Nelder–Mead simplex. For each shape, the fit for the previous stage of inversion was used as the initial guess for the optimisation. Sample code is given in S2 Code.

### Shape perturbations and statistical statements

To define perturbations for the *F* = 15 fitted model parameters ***P***_**0**_ ∈ ℝ^*F*^ at noise level *δ*, we draw independent *N* uniform random samples $X∼U{\left[0,1\right]}^{F}$ on the unit interval and define the perturbed parameters ***P*** = ***P***_**0**_(1 − *δ* + 2*δ****X***) by pointwise multiplication.

#### Uniformity of the distribution of perturbations

A caveat applies to the computational method: if the relative size of perturbations (the 'noise level') exceeds about 4% at the stage of inversion discussed in the main text, computation of the perturbed shapes fails for some parameter choices. This mechanical effect is not surprising: our previous analysis of invagination [56] revealed strong shape nonlinearities and the possibility of bifurcations as the magnitude of the intrinsic curvature in the bend region is increased. While we may therefore expect more leeway in some parameters than in others, we shall simply discard those perturbations for which the computation fails; further estimation of the distribution of possible perturbations is beyond the scope of the present discussion.

At the noise level of 7.5% appropriate for our analysis, about 15% of perturbations fail; the resulting nonuniformities are small but statistically significant. Indeed, the samples that are retained are uniform on an unknown set $A\subseteq {\left[0,1\right]}^{F}$ with means ***μ***. To establish that these means are not all the same, we derive confidence intervals for *μ*_*i*_ − *μ*_*j*_. Since |*X*_*i*_ − *X*_*j*_| ⩽ 1, we may bound the variance of these differences by Var(*X*_*i*_ − *X*_*j*_) ⩽ 1, and hence, by the central limit theorem, a 100(1−*p*)% confidence interval is

$$\langle X_{i}\rangle -\langle X_{j}\rangle \pm \frac{z}{\sqrt{N}},\;where\;z=\Phi^{-1}\left(1-\frac{p/2}{\left(\begin{array}{c} F\\ 2 \end{array}\right)}\right),$$ (38)

wherein Φ^−1^ is the inverse of the cumulative distribution function of the N(0,1) distribution and wherein we have included a multiple-testing correction. At noise level *δ* = 0.075, we have run *N* = 10,000 perturbations (S2 Data), finding *M* = max⟨***X***⟩ ≈ 0.526 and *m* = min⟨***X***⟩ ≈ 0.485. With *M*−*m* ≈ 0.041 and $\Phi^{-1}(1-0.005/105)/\sqrt{N}\approx 0.039$, we infer that the 99% confidence interval for the maximum difference of the means does not contain zero and hence that the means are not all the same. We notice, however, that these deviations of the means are small in that they are not statistically significantly different from 0.5.

#### Position of the maxima of shape variation

We now make quantitative our statement, based on the cumulative distributions in Fig 11C, that the experimental distribution of shape variation (with a maximum in the anterior fold) is very unlikely to arise under the uniform model. We ask: what is the probability *p*, under the uniform model, for the maximum in shape variation to lie in the anterior fold (Fig 11C)? For 10,000 perturbations (S2 Data), we found that 757 had a maximum in the anterior fold. Among these perturbations, 2,345 yielded a single maximum in shape variation, with 60 of these maxima in the anterior fold. With 99% confidence, we therefore have upper bounds *p* < 0.0757 + 0.0129 < 0.09 from all perturbations, and *p* < 0.0256 + 0.0266 < 0.06 if we restrict to shape variations with a single maximum.

## Supporting information

S1 Fig Cell movement relative to cytoplasmic bridges. A motor protein, the kinesin InvA, is associated with cortical microtubules and an unknown structure within the cytoplasmic bridges in *V*. *carteri* [54]. As the cells in the bend region develop think stalks, InvA 'walks' towards the plus end of the microtubules, moving the cells until they are connected at the tips of their stalks.
(TIF)

S2 Fig Alternative averaging approach 1. Alignment of embryos by the timepoint during which the posterior-to-bend distance *e* reaches half of its initial value, without time stretching. *N* = 22 overlaid and scaled embryo halves from experimental data (lines in shades of blue), and averages thereof (red lines), for 10 stages of inversion. Shaded areas correspond to standard deviation shapes. At late-inversion stages, the average shapes are very noisy. See S1 Data for numerical values.
(TIF)

S3 Fig Alternative averaging approach 2. Alignment of embryos with time stretching and with uniformly distributed averaging points (i.e., with only global scaling of embryos, without relative local stretching of embryo shapes). *N* = 22 overlaid and scaled embryo halves from experimental data (lines in shades of blue), and averages thereof (red lines), for 10 stages of inversion. Shaded areas correspond to standard deviation shapes. Unsatisfactory 'kinks' arise in the bend region. See S1 Data for numerical values.
(TIF)

S4 Fig Comparison of mean shape variation for different averaging methods. Mean shape variation against mean time ⟨*t*⟩ for the three averaging methods in Fig 4, S2 Fig, and S3 Fig, showing that the averaging method using time stretching and local relative stretching of embryo shapes yields better averages than the two alternative averaging methods, especially at mid- to late-inversion stages. For alignment by posterior-to-bend distance, mean time was determined approximately by comparing the shapes in Fig 4, S2 Fig, S3 Fig. See S1 Data for numerical values.
(TIF)

S1 Video Inversion in *V*. *globator*. Time-lapse video of inverting *V*. *globator* embryo from selective plane illumination imaging of chlorophyll autofluorescence. Left: maximum intensity projection of z-stacks. Right: tracing of midsagittal cross-section (Materials and methods). Scale bar: 50 μm.
(MP4)

S2 Video Cell rearrangement at the phialopore. Time-lapse video of the phialopore opening obtained from confocal laser scanning microscopy of chlorophyll autofluorescence and manual tracing of selected cells (Materials and methods). Scale bar: 20 μm. The video shows a rearrangement of cells surrounding the phialopore.
(MP4)

S1 Data Numerical data. Numerical values underlying the shapes and graphs shown in Figs 4, 5, 7, 8 and 11 in the main text; supplementary figures S2 Fig, S3 Fig, S4 Fig, and figures A1, A2, A3, A4 in S1 Text. Numerical values of the fitting parameters used to obtain Fig 7. Additional data for analysis of variability.
(XLSX)

S2 Data Raw data for random perturbations. Random perturbations of parameters, corresponding shape variations, and other statistics used for the analysis of variability.
(GZ) Click here for additional data file.

S1 Code Code for tracing embryo shapes. Elements of Python code used for semiautomated embryo shape tracing.
(GZ) Click here for additional data file.

S2 Code Code for numerical calculations. Elements of Matlab (The MathWorks) code used for numerical solution of the equations governing the model, for aligning shapes, and for fitting shapes.
(M) Click here for additional data file.

S1 Text Summary statistics and geometric descriptors of inversion. Initial analysis of the variability using summary statistics. Analysis of inversion in terms of six geometric descriptors and comparison of geometric descriptors for averaged and fitted shapes.
(PDF)

S2 Text Elastic model in the contact configuration. Boundary conditions for the contact configuration. Numerical study of the contact configuration. Asymptotic analysis of a toy problem.
(PDF)

## References

[1] Bichat MFX. Recherches physiologiques sur la vie et la mort. Paris, France: Brosson, Gabon et Compagnie; An VIII.

[2] Hong L, Dumond M, Tsugawa S, Sapala A, Routier-Kierzkowska AL, Zhou Y, et al Variable Cell Growth Yields Reproducible Organ Development through Spatiotemporal Averaging. Dev Cell. 2016;38(1):15–32. 10.1016/j.devcel.2016.06.016 27404356

[3] Lecuit T, Lenne PF. Cell surface mechanics and the control of cell shape, tissue patterns and morphogenesis. Nat Rev Mol Cell Biol. 2007;8:633–644. 10.1038/nrm2222 17643125

[4] Lecuit T, Lenne PF, Munro E. Force generation, transmission, and integration during cell and tissue morphogenesis. Annu Rev Cell Dev Bi. 2011;27:157–184. 10.1146/annurev-cellbio-100109-104027 21740231

[5] Sweeton D, Parks S, Costa M, Wieschaus E. Gastrulation in *Drosophila*: the formation of the ventral furrow and posterior midgut invaginations. Development. 1991;112:775–789. 1935689 10.1242/dev.112.3.775

[6] Urbansky S, González Avalos P, Wosch M, Lemke S. Folded gastrulation and T48 drive the evolution of coordinated mesoderm internalization in flies. eLife. 2016;5:e18318 10.7554/eLife.18318 27685537 PMC5042651

[7] Leptin M. Gastrulation movements: the logic and the nuts and bolts. Dev Cell. 2005;8:305–320. 10.1016/j.devcel.2005.02.007 15737927

[8] Wang Y, Steinbeisser H. Molecular basis of morphogenesis during vertebrate gastrulation. Cell Mol Life Sci. 2009;66:2263–2273. 10.1007/s00018-009-0018-2 19347571 PMC11115717

[9] Fuhrmann S. Eye morphogenesis and patterning of the optic vesicle. Curr Top Dev Biol. 2010;93:61–84. 10.1016/B978-0-12-385044-7.00003-5 20959163 PMC2958684

[10] Chauhan B, Plagman T, Lou M, Lang R. Epithelial morphogenesis: the mouse eye as a model system. Curr Top Dev Biol. 2015;111:375–399. 10.1016/bs.ctdb.2014.11.011 25662266 PMC6014593

[11] Lowery LA, Sive H. Strategies of vertebrate neurulation and a re-evaluation of teleost neural tube formation. Mech Dev. 2004;121:1189–1197. 10.1016/j.mod.2004.04.022 15327780

[12] Vijayraghavan DS, Davidson LA. Mechanics of neurulation: from classical to current perspectives on the physical mechanics that shape, fold, and form the neural tube. Birth Defects Res. 2017;109:153–168. 10.1002/bdra.23557 27620928 PMC9972508

[13] Sherrard K, Robin F, Lemaire P, Munro E. Sequential activation of apical and basolateral contractility drives ascidian endoderm invagination. Curr Biol. 2010;20(17):1499–1510. 10.1016/j.cub.2010.06.075 20691592 PMC4088275

[14] Sasai Y, Eiraku M, Suga H. *In vitro* organogenesis in three dimensions: self-organising stem cells. Development. 2012;139:4111–4121. 10.1242/dev.079590 23093423

[15] Odell GM, Oster G, Alberch P, Burnside B. The mechanical basis of morphogenesis. Dev Biol. 1981;85(2):446–462. 10.1016/0012-1606(81)90276-1 7196351

[16] Hardin JD, Cheng LY. The mechanisms and mechanics of archenteron elongation during sea urchin gastrulation. Dev Biol. 1986;115(2):490–501. 10.1016/0012-1606(86)90269-1

[17] Hardin J, Keller R. The behaviour and function of bottle cells during gastrulation of *Xenopus laevis*. Development. 1988;103:211–230. 3197630 10.1242/dev.103.1.211

[18] Davidson LA, Koehl MA, Keller R, Oster GF. How do sea urchins invaginate? Using biomechanics to distinguish between mechanisms of primary invagination. Development. 1995;121(7):2005–2018. 7635048 10.1242/dev.121.7.2005

[19] Davidson LA, Oster GF, Keller RE, Koehl MAR. Measurements of Mechanical Properties of the Blastula Wall Reveal Which Hypothesized Mechanisms of Primary Invagination Are Physically Plausible in the Sea Urchin *Strongylocentrotus purpuratus*. Dev Biol. 1999;209(2):221–238. 10.1006/dbio.1999.9249 10328917

[20] Fletcher AG, Cooper F, Baker RE. Mechanocellular models of epithelial morphogenesis. Phil Trans Roy Soc B. 2017;372 10.1098/rstb.2015.0519 28348253 PMC5379025

[21] Rauzi M, Hočevar Brezavšček A, Ziherl P, Leptin M. Physical Models of Mesoderm Invagination in *Drosophila* Embryo. Biophys J. 2013;105:3–10. 10.1016/j.bpj.2013.05.039 23823218 PMC3699736

[22] Howard J, Grill SW, Blois JS. Turing's next steps: the mechanochemical basis of morphogenesis. Nat Rev Mol Cell Biol. 2011;12:392–398. 10.1038/nrm3120 21602907

[23] Prost J, Jülicher F, Joanny JF. Active gel physics. Nat Phys. 2015;11:111–117. 10.1038/nphys3224

[24] Cooper WJ, Albertson RC. Quantification and variation in experimental studies of morphogenesis. Dev Biol. 2008;321(2):295–302. 10.1016/j.ydbio.2008.06.025 18619435

[25] Oates AC, Gorfinkel N, González-Gaitán M, Heisenberg CP. Quantitative approaches in developmental biology. Nature Rev Gen. 2009;10:517–530. 10.1038/nrg2548 19584811

[26] von Dassow M, Davidson LA. Variation and Robustness of the Mechanics of Gastrulation: The Role of Tissue Mechanical Properties During Morphogenesis. Birth Defects Res. 2007;81:253–269. 10.1002/bdrc.20108 18228257

[27] Cherdantsev VG, Tsvetkova NV. Dynamics and Variability of Early Morphogenesis in the Loach according to Observations of Individual Developmental Trajectories. Russ J Dev Biol. 2005;36(3):171–180. 10.1007/s11174-005-0027-5 15977804

[28] Cherdantsev VG, Korvin-Pavlovskaya EG. Variability of quantitative morphogenetic parameters during early morphogenesis of the loach, *Misgurnus fossilis L*. Russ J Dev Biol. 2016;47(1):49–62. 10.1134/S1062360416010021 27149749

[29] Huxley J. The Individual in the Animal Kingdom. Cambridge, United Kingdom: Cambridge University Press; 1912.

[30] Weismann A. Essays on heredity and kindred biological problems Oxford, United Kingdom: Clarendon Press; 1892.

[31] Kirk DL. *Volvox*: molecular-genetic origins of multicellularity and cellular differentiation. Cambridge, United Kingdom: Cambridge University Press; 1998.

[32] Kirk DL. A twelve-step program for evolving multicellularity and a division of labor. BioEssays. 2005;27(3):299–310. 10.1002/bies.20197 15714559

[33] Herron MD. Origins of multicellular complexity: *Volvox* and the volvocine algae. Mol Ecol. 2016;25(6):1213–1223. 10.1111/mec.13551 26822195 PMC5765864

[34] Goldstein RE. Green Algae as Model Organisms for Biological Fluid Dynamics. Annu Rev Fluid Mech. 2015;47:343–375. 10.1146/annurev-fluid-010313-141426 26594068 PMC4650200

[35] Kirk DL, Birchem R, King N. The extracellular matrix of *Volvox*: a comparative study and proposed system of nomenclature. J Cell Sci. 1986;80(1):207–231. 3722281 10.1242/jcs.80.1.207

[36] Hallmann A. Extracellular Matrix and Sex-Inducing Pheromone in *Volvox*. Int Rev Cytol. 2003;227:131–182. 10.1016/S0074-7696(03)01009-X 14518551

[37] Green KJ, Kirk DL. Cleavage patterns, cell lineages, and development of a cytoplasmic bridge system in *Volvox* embryos. J Cell Biol. 1981;91(3):743–755. 10.1083/jcb.91.3.743 7328119 PMC2112818

[38] Green KJ, Viamontes GI, Kirk DL. Mechanism of formation, ultrastructure, and function of the cytoplasmic bridge system during morphogenesis in *Volvox*. J Cell Biol. 1981;91(3):756–769. 10.1083/jcb.91.3.756 7328120 PMC2112823

[39] Hoops HJ, Nishii I, Kirk DL. Cytoplasmic Bridges in Volvox and Its Relatives In: Baluska F, Volkmann D, Barlow PW, editors. Cell-Cell Channels. New York, NY: Springer; 2006 p. 65–84.

[40] Kirk DL, Viamontes GI, Green KJ, Bryant JL Jr. Integrated Morphogenetic Behavior of Cell Sheets: *Volvox* as a Model In: Subtelny S, Green PB, editors. Developmental Order: Its Origin and Regulation. New York, NY: Alan R. Liss; 1982 p. 247–274.

[41] Kirk DL, Nishii I. *Volvox carteri* as a model for studying the genetic and cytological control of morphogenesis. Dev Growth Differ. 2001;43(6):621–631. 10.1046/j.1440-169X.2001.00612.x 11737143

[42] Matt G, Umen J. *Volvox*: A simple algal model for embryogenesis, morphogenesis and cellular differentiation. Dev Biol. 2016;419(1):99–113. 10.1016/j.ydbio.2016.07.014 27451296 PMC5101179

[43] Höhn S, Honerkamp-Smith AR, Haas PA, Khuc Trong P, Goldstein RE. Dynamics of a *Volvox* Embryo Turning Itself Inside Out. Phys Rev Lett. 2015;114:178101 10.1103/PhysRevLett.114.178101 25978266

[44] Höhn S, Hallmann A. There is more than one way to turn a spherical cellular monolayer inside out: type B embryo inversion in *Volvox globator*. BMC Biol. 2011;9:89 10.1186/1741-7007-9-89 22206406 PMC3324393

[45] Viamontes GI, Kirk DL. Cell shape changes and the mechanism of inversion in *Volvox*. J Cell Biol. 1977;75(3):719–730. 10.1083/jcb.75.3.719 925078 PMC2111588

[46] Hallmann A. Morphogenesis in the Family Volvocaceae: Different Tactics for Turning an Embryo Right-side Out. Protist. 2006;157(4):445–461. 10.1016/j.protis.2006.05.010 16854623

[47] Iida H, Nishii I, Inouye I. Embryogenesis and cell positioning in *Platydorina caudata* (Volvocaceae, Chlorophyta). Phycologia. 2011;50(5):530–540. 10.2216/10-80.1

[48] Iida H, Ota S, Inouye I. Cleavage, incomplete inversion, and cytoplasmic bridges in *Gonium pectorale* (Volvocales, Chlorophyta). J Plant Res. 2013;126:699–707. 10.1007/s10265-013-0553-7 23455615

[49] Höhn S, Hallmann A. Distinct shape-shifting regimes of bowl-shaped cell sheets–embryonic inversion in the multicellular green alga *Pleodorina*. BMC Dev Biol. 2016;16:35 10.1186/s12861-016-0134-9 27733125 PMC5062935

[50] Zimmermann W. Die ungeschlechtliche Entwicklung von *Volvox*. Naturwissenschaften. 1925;13(19):397–402. 10.1007/BF01560949

[51] Keller R, Shook D. The bending of cell sheets—from folding to rolling. BMC Biol. 2011;9:90 10.1186/1741-7007-9-90 22206439 PMC3248374

[52] Feroze R, Shawky JH, von Dassow M, Davidson LA. Mechanics of blastopore closure during amphibian gastrulation. Dev Biol. 2015;398:57–67. 10.1016/j.ydbio.2014.11.011 25448691 PMC4317491

[53] Czerniak ND, Dierkes K, D'Angelo A, Colombelli J, Solon J. Patterned Contractile Forces Promote Epidermal Spreading and Regulate Segment Positioning during Drosophila Head Involution. Curr Biol. 2016;26(14):1895–1901. 10.1016/j.cub.2016.05.027 27397891

[54] Nishii I, Ogihara S, Kirk DL. A kinesin, InvA, plays an essential role in *Volvox* morphogenesis. Cell. 2003;113:743–753. 10.1016/S0092-8674(03)00431-8 12809605

[55] Nishii I, Ogihara S. Actomyosin contraction of the posterior hemisphere is required for inversion of the *Volvox* embryo. Development. 1999;126(10):2117–2127. 10207137 10.1242/dev.126.10.2117

[56] Haas PA, Goldstein RE. Elasticity and Glocality: Initiation of Embryonic Inversion in *Volvox*. J R Soc Interface. 2015;12:20150671 10.1098/rsif.2015.0671 26490631 PMC4685841

[57] Goriely A. In: The Mathematics and Mechanics of Biological Growth. Berlin, Germany: Springer; 2017 p. 345–373 & 555–579.

[58] Audoly B, Pomeau Y. Elasticity and Geometry: From Hair Curls to the Non-linear Response of Shells. Oxford, United Kingdom: Oxford University Press; 2010.

[59] Libai A, Simmonds JG. The Nonlinear Elasticity of Elastic Shells. 2nd ed Cambridge, United Kingdom: Cambridge University Press; 2005.

[60] Pitrone PG, Schindelin J, Stuyvenberg L, Preibisch S, Weber M, Eliceiri KW, et al OpenSPIM: an open-access light-sheet microscopy platform. Nat Methods. 2013;10:598–599. 10.1038/nmeth.2507 23749304 PMC7450513

[61] Thompson DW. In: On Growth and Form. 2nd ed Cambridge, United Kingdom: Cambridge University Press; 1941 p. 1026–1092.

[62] Viamontes GI, Fochtmann LJ, Kirk DL. Morphogenesis in *Volvox*: analysis of critical variables. Cell. 1979;17:537–550. 10.1016/0092-8674(79)90262-9 476832

[63] Kelland JL. Inversion in *Volvox* (Chlorophyceae). J Phycol. 1977;13(4):373–378. 10.1111/j.1529-8817.1977.tb02945.x

[64] Houchmandzadeh B, Wieschaus E, Leibler S. Precise domain specification in the developing *Drosophila* embryo. Phys Rev E. 2005;72(6):061920 10.1103/PhysRevE.72.061920 16485987

[65] Turing AM. The chemical basis of morphogenesis. Phil Trans Roy Soc B. 1952;237:37–72. 10.1098/rstb.1952.0012

[66] Houchmandzadeh B, Wieschaus E, Leibler S. Establishment of developmental precision and proportions in the early *Drosophila* embryo. Nature. 2002;415:798–802. 10.1038/415798a 11845210

[67] Keller R, Davidson LA, Shook DR. How we are shaped: The biomechanics of gastrulation. Differentiation. 2003;71(3):171–205. 10.1046/j.1432-0436.2003.710301.x 12694202

[68] Goldstein RE. Batchelor Prize Lecture: Fluid dynamics at the scale of the cell. J Fluid Mech. 2016;807:1–39. 10.1017/jfm.2016.586

[69] Dasbiswas K, Alster E, Safran SA. Mechanobiological induction of long-range contractility by diffusing biomolecules and size scaling in cell assemblies. Sci Rep. 2016;6:27692 10.1038/srep27692 27283037 PMC4901349

[70] Hannezo E, Prost J, Joanny JF. Theory of epithelial sheet morphology in three dimensions. Proc Nat Acad Sci USA. 2014;111(1):27–32. 10.1073/pnas.1312076111 24367079 PMC3890844

[71] Dasbiswas K, Hannezo E, Gov NS. Theory of Epithelial Cell Shape Transitions Induced by Mechanoactive Chemical Gradients. Biophys J. 2018;114(4):968–977. 10.1016/j.bpj.2017.12.022 29490256 PMC5984993

[72] Bhalerao RP, Bennett MJ. The case for morphogens in plants. Nat Cell Biol. 2003;5(11):939–943. 10.1038/ncb1103-939 14593411

[73] Bickerton PD, Pittman JK. Calcium Signalling in Plants. In: eLs. Chichester: John Wiley & Sons; 2012.

[74] Fujiu K, Nakayama Y, Iida H, Sokabe M, Yoshimura K. Mechanoreception in motile flagella of *Chlamydomonas*. Nat Cell Biol. 2011;13(5):630–632. 10.1038/ncb2214 21478860

[75] Pickett-Heaps JD. Some ultrastructural features of *Volvox*, with particular reference to the phenomenon of inversion. Planta. 1970;90(2):174–190. 10.1007/BF00388045 24500745

[76] Abkarian M, Massiera G, Berry L, Roques M, Braun-Breton C. A novel mechanism for egress of malarial parasites from red blood cells. Blood. 2011;117(15):4118–4124. 10.1182/blood-2010-08-299883 21297002

[77] Kabaso D, Shlomovitz R, Auth T, Lew VL, Gov NS. Curling and Local Shape Changes of Red Blood Cell Membranes Driven by Cytoskeletal Reorganization. Biophys J. 2010;99(3):808–816. 10.1016/j.bpj.2010.04.067 20682258 PMC2913190

[78] Callan-Jones A, Albarran Arriagada OE, Massiera G, Abkarian VLM. Red Blood Cell Membrane Dynamics during Malaria Parasite Egress. Biophys J. 2012;103(12):2475–2483. 10.1016/j.bpj.2012.11.008 23260049 PMC3525858

[79] Yamashita S, Arakaki Y, Kawai-Toyooka H, Noga A, Hirono M, Nozaki H. Alternative evolution of a spheroidal colony in volvocine algae: developmental analysis of embryogenesis in *Astrephomene* (Volvocales, Chlorophyta). BMC Evol Biol. 2016;16:243 10.1186/s12862-016-0794-x 27829356 PMC5103382

[80] Short MB, Solari CA, Ganguly S, Powers TR, Kessler JO, Goldstein RE. Flows driven by flagella of multicellular organisms enhance long-range molecular transport. Proc Nat Acad Sci USA. 2006;103(22):8315–8319. 10.1073/pnas.0600566103 16707579 PMC1482491

[81] Herron MD, Desnitskiy AG, Michod RE. Evolution of Developmental Programs in *Volvox* (Chlorophyta). J Phycol. 2010;46(2):316–324. 10.1111/j.1529-8817.2009.00803.x

[82] Herron MD, Michod RE. Evolution of Complexity in the Volvocine Algae: Transitions in Individuality through Darwin's eye. Evolution. 2008;62(2):436–451. 10.1111/j.1558-5646.2007.00304.x 18031303

[83] Pocock MA. *Volvox* in South Africa. Ann S Afr Mus. 1933;16:523–646.

[84] von Dassow M, Davidson LA. Physics and the canalization of morphogenesis: a grand challenge in organismal biology. Phys Biol. 2011;8(4):045002 10.1088/1478-3975/8/4/045002 21750364 PMC3200556

[85] Schlösser UG. SAG–Sammlung von Algenkulturen at the University of Göttingen, Catalogue of Strains 1994. Bot Acta. 1994;107(3):113–186. 10.1111/j.1438-8677.1994.tb00784.x

[86] Brumley DR, Wan KY, Polin M, Goldstein RE. Flagellar synchronization through direct hydrodynamic interactions. eLife. 2014;3:e02750 10.7554/eLife.02750 25073925 PMC4113993

[87] Schindelin J, Arganda-Carreras I, Frise E, Kaynig V, Longair M, Pietzsch T, et al Fiji: an open-source platform for biological-image analysis. Nat Methods. 2012;9:676–682. 10.1038/nmeth.2019 22743772 PMC3855844

[88] Kass M, Witkin A, Terzopoulos D. Snakes: Active Contour Models. Int J Comput Vision. 1988;1(4):312–331. 10.1007/BF00133570

[89] Zhang TY, Suen CY. A fast parallel algorithm for thinning digital patterns. Commun ACM. 1984;27(3):236–239. 10.1145/357994.358023

[90] Otsu N. A threshold selection method from gray-level histograms. IEEE T Syst Man Cyb. 1979;9(1):62–66. 10.1109/TSMC.1979.4310076

[91] Müller M. Dynamic Time Warping In: Information Retrieval for Music and Motion. Berlin, Germany: Springer; 2007 p. 69–84.

[92] Gao F, Han L. Implementing the Nelder–Mead simplex algorithm with adaptive parameters. Comput Optim Appl. 2012;51:259–277. 10.1007/s10589-010-9329-3

[93] Knoche S, Kierfeld J. Buckling of Spherical Capsules. Phys Rev E. 2011;84:046608 10.1103/PhysRevE.84.046608 22181297

[94] Efrati E, Sharon E, Kupferman R. Elastic theory of unconstrained non-Euclidean plates. J Mech Phys Solids. 2009;57(4):762–775. 10.1016/j.jmps.2008.12.004

