# Supplementary figures and images for: The noisy basis of morphogenesis: Mechanisms and mechanics of cell sheet folding inferred from developmental variability
Source: PLoS Biol. 2018 Jul 12;16(7):e2005536. doi: 10.1371/journal.pbio.2005536 (PMC6063725; doi:10.1371/journal.pbio.2005536)

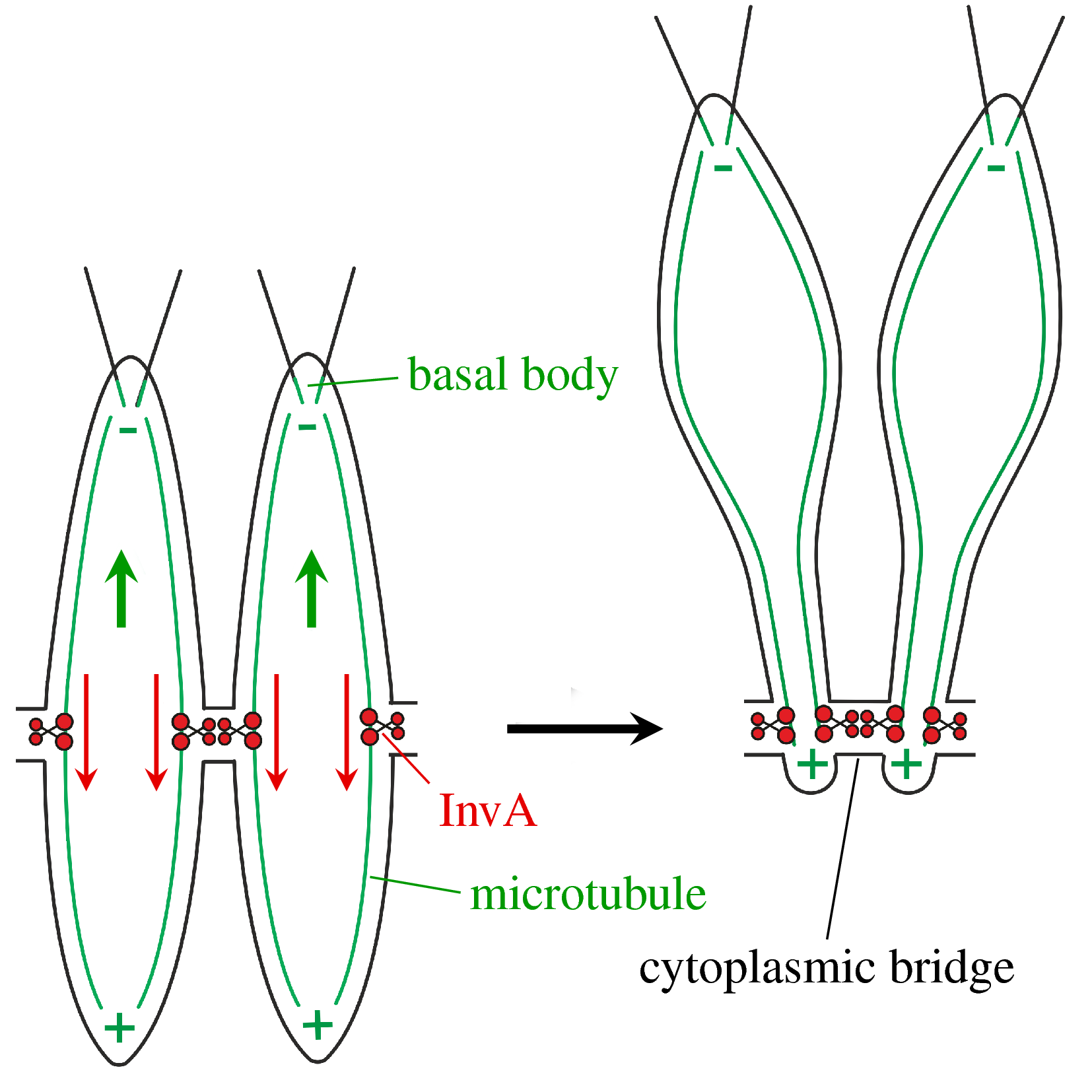

Supplement: S1 Fig — A motor protein, the kinesin InvA, is associated with cortical microtubules and an unknown structure within the cytoplasmic bridges in V. carteri [54]. As the cells in the bend region develop think stalks, InvA 'walks' towards the plus end of the microtubules, moving the cells until they are connected at the tips of their stalks. (TIF) [file pbio.2005536.s001.tif]

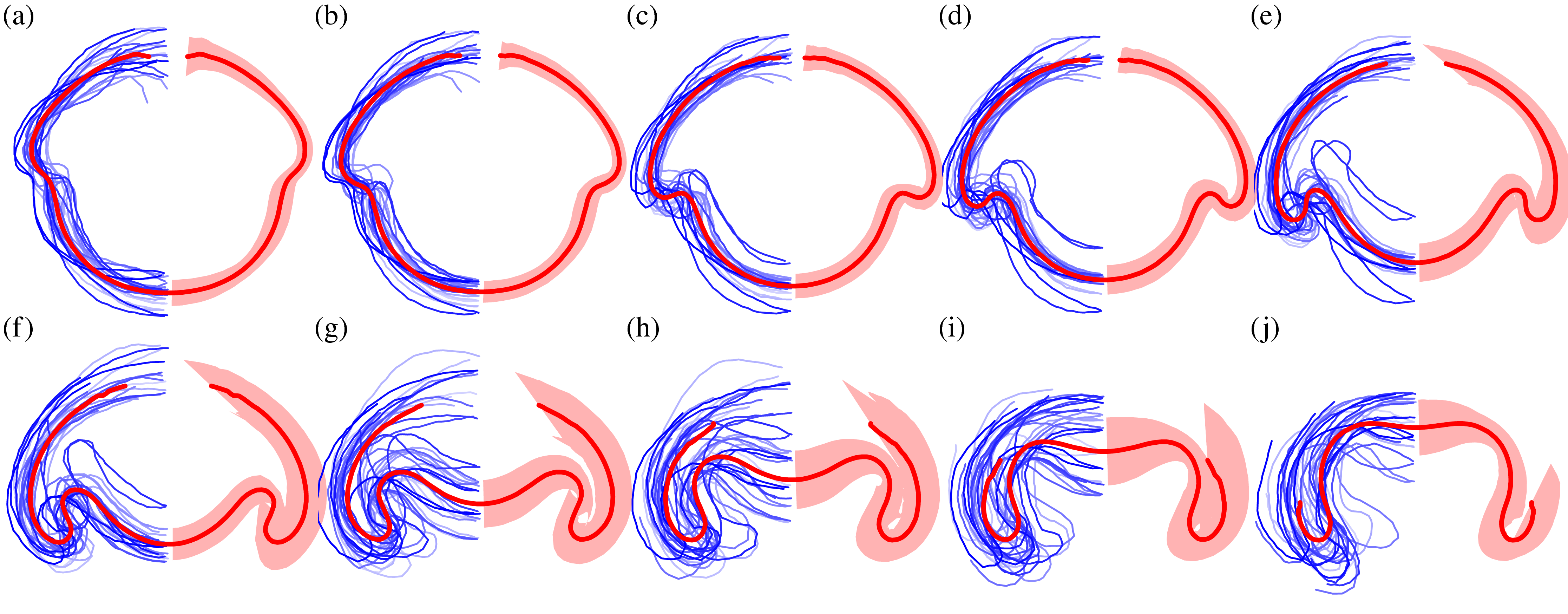

Supplement: S2 Fig — Alignment of embryos by the timepoint during which the posterior-to-bend distance e reaches half of its initial value, without time stretching. N = 22 overlaid and scaled embryo halves from experimental data (lines in shades of blue), and averages thereof (red lines), for 10 stages of inversion. Shaded areas correspond to standard deviation shapes. At late-inversion stages, the average shapes are very noisy. See S1 Data for numerical values. (TIF) [file pbio.2005536.s002.tif]

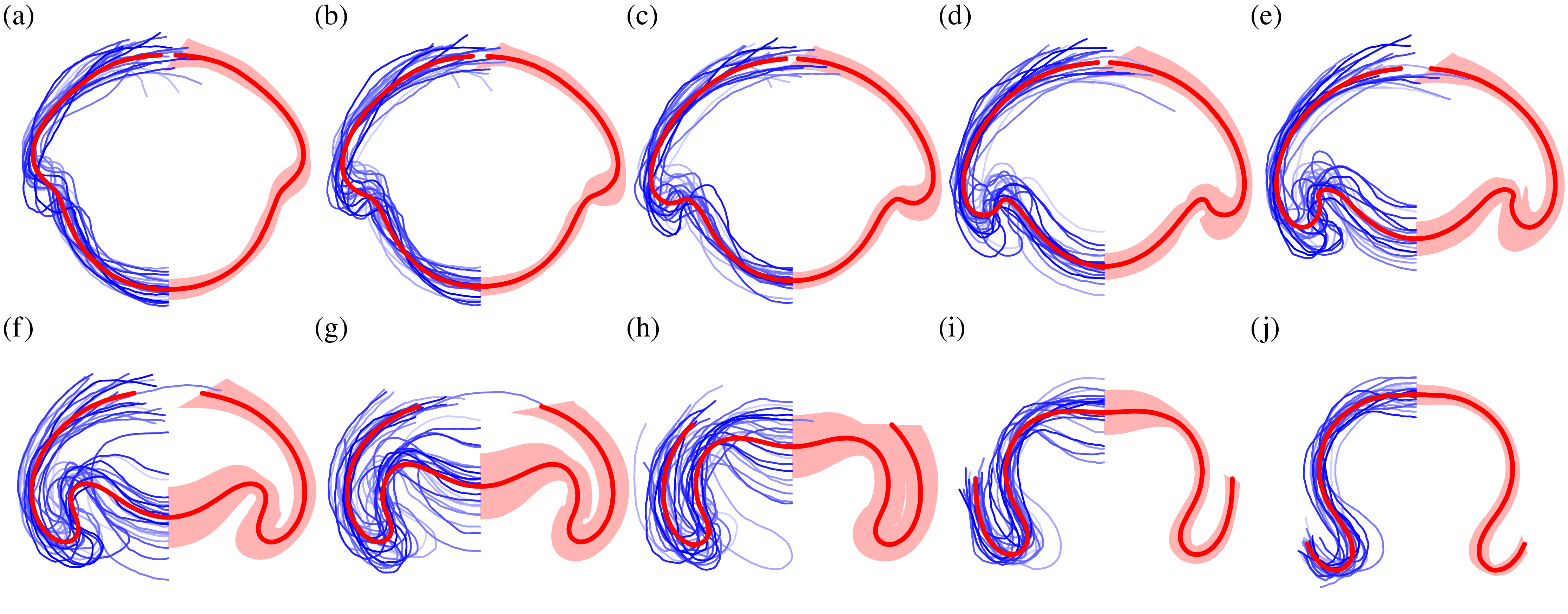

Supplement: S3 Fig — Alignment of embryos with time stretching and with uniformly distributed averaging points (i.e., with only global scaling of embryos, without relative local stretching of embryo shapes). N = 22 overlaid and scaled embryo halves from experimental data (lines in shades of blue), and averages thereof (red lines), for 10 stages of inversion. Shaded areas correspond to standard deviation shapes. Unsatisfactory 'kinks' arise in the bend region. See S1 Data for numerical values. (TIF) [file pbio.2005536.s003.tif]

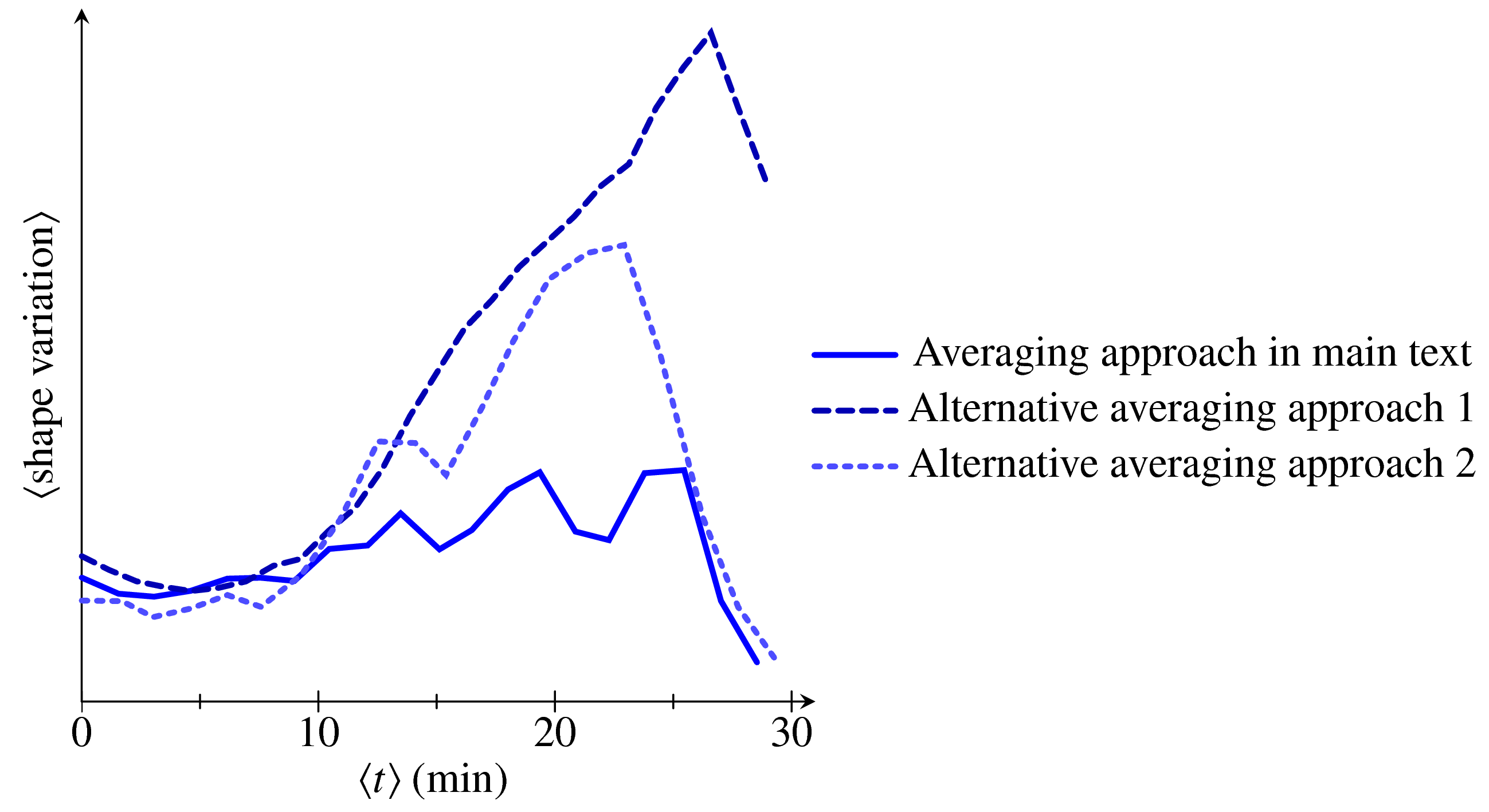

Supplement: S4 Fig — Mean shape variation against mean time ⟨t⟩ for the three averaging methods in Fig 4, S2 Fig, and S3 Fig, showing that the averaging method using time stretching and local relative stretching of embryo shapes yields better averages than the two alternative averaging methods, especially at mid- to late-inversion stages. For alignment by posterior-to-bend distance, mean time was determined approximately by comparing the shapes in Fig 4, S2 Fig, S3 Fig. See S1 Data for numerical values. (TIF) [file pbio.2005536.s004.tif]
