# Supplementary material for: The noisy basis of morphogenesis: Mechanisms and mechanics of cell sheet folding inferred from developmental variability
Source: PLoS Biol. 2018 Jul 12;16(7):e2005536. doi: 10.1371/journal.pbio.2005536 (PMC6063725; doi:10.1371/journal.pbio.2005536)
Supplement: S1 Text — Initial analysis of the variability using summary statistics. Analysis of inversion in terms of six geometric descriptors and comparison of geometric descriptors for averaged and fitted shapes. (PDF) [file pbio.2005536.s011.pdf]

The noisy basis of morphogenesis: mechanisms and mechanics of cell sheet folding inferred from developmental variability

P. A. Haas, S. S. M. H. Höhn, A. R. Honerkamp-Smith, J. B. Kirkegaard, and R. E. Goldstein

S1 Text. Summary Statistics and Geometric Descriptors of Inversion.

An initial impression of the variability of inversion is provided by the analysis of three summary statistics: (1) the duration of inversion, from appearance of the bend region to closure of the phialopore, (2) the diameter of the embryos post-inversion, (3) the relative time during inversion at which the phialopore starts to open. Histograms of these quantities in Fig. A1a–c reveal considerable variability, thus showing that the noise does not only affect the global duration of inversion, but also the relative timing of parts of it. We additionally note that there is no correlation between the size of an embryo and the duration of its inversion (Fig. A1d), not even between embryos from the same parent spheroid.

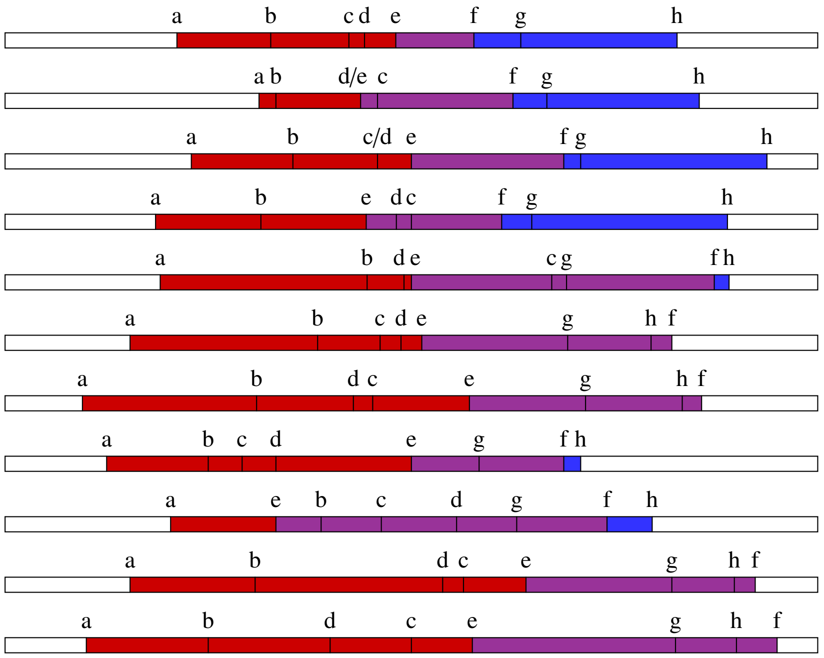

Figure A1. Summary Statistics for Inversion from $N = 33$ Embryos. Histograms of (a) duration of inversion, (b) embryo diameter (after inversion), and (c) relative time of phialopore opening. (d) Duration of inversion plotted against embryo diameter (after inversion). Data points corresponding to embryos from the same parent spheroid are shown in the same colour. See S1 Data for numerical values.

It is natural to ask to what extent the different deformations of inversion must arise in a particular order: while invagination occurs before phialopore opening in all our samples, analysis of characteristic ‘checkpoints’ of inversion (Fig. A2) reveals that there is still considerable leeway in the timing of posterior inversion and phialopore opening. This is the same non-linearity that we inferred more generally in Fig. 5a from the global shapes, with some embryos lingering in some stages of inversion.

In our previous work [43], we discussed in detail three geometric descriptors of the traced embryo outlines, which we have reproduced for the present dataset:

1. the distance e (Fig. A3a) from the posterior pole to the plane of the circular bend region; this serves as an indicator of the progress of the ‘upwards’ movement of the posterior hemisphere;

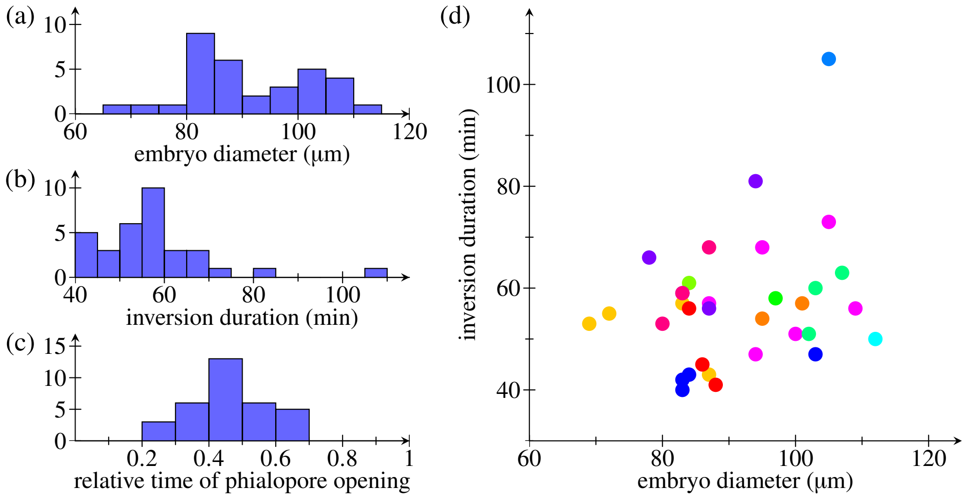

Figure A2. Timelines showing the variability of characteristic points of the measurements in Fig. A3, normalised by the total duration of inversion from appearance of the bend region until closure of the phialopore. a: first measurement of posterior-to-bend distance e (when the tangent at the point of the most negative curvature κ_* is horizontal); b: maximal negative curvature κ_*; c: maximal surface area A; d: e reaches half of its initial value; e: phialopore has widened to 20% of its maximal diameter, f: e reaches 10% of its initial value; g: phialopore reaches its maximal diameter; h: phialopore has shrunk to 20% of its maximal diameter. Posterior inversion (characteristic points a,d,f) is shown in red, and anterior inversion (characteristic points e,g,h) is shown in blue. The purple regions indicate an overlap of posterior and anterior inversion. See S1 Data for numerical values.

2. the embryonic surface area A (Fig. A3b), which was computed by determining a surface of revolution from each half of the midsagittal slice and averaging the two values for each timepoint;

3. the minimal (most negative) value κ_* of the meridional curvature in the bend region (Fig. A3c).

We have computed three additional descriptors associated with the progress of later of inversion:

4. the diameter d of the phialopore (Fig. A3d) as an indicator of progress of inversion of the anterior hemisphere;

5. the width w of the bend region (Fig. A3e), where the bend region is defined as the region of negative curvature;

6. the position of the bend region (Fig. A3f), measured along the arclength of the deformed shell from the posterior pole to the midpoint of the bend region.

From the aligned shapes, these geometric descriptors were computed as follows: the posterior-to-bend distance e was computed as the distance from the apex line to the posterior pole. The maximal surface area A_{max} and the most negative value of curvature κ_* in the bend region were computed as described previously [43]; traces were smoothed before computing the curvature. The phialopore width d was computed as the absolute distance between the two ends of a complete embryo trace. The bend region was defined as the region of negative curvature; the distance between the first and last points of negative curvature defined the bend region width. The bend region position is defined by the distance, along the embryo trace, between the posterior pole and the midpoint of the bend region. (The latter may differ from the point where the most negative value of curvature is attained.) The values of bend region width and position obtained for each embryo half were averaged to yield the reported values.

While the posterior hemisphere moves into the anterior hemisphere (Fig. A3a), the surface area increases considerably due to stretching in the anterior hemisphere [43], in most embryos before the phialopore begins to widen (Fig. A3b,d). While the negative curvature in the bend region increases, the meridional width of the bend region widens (Fig. A3c,e) and the distance of its midpoint to the posterior pole decreases (Fig. A3f).

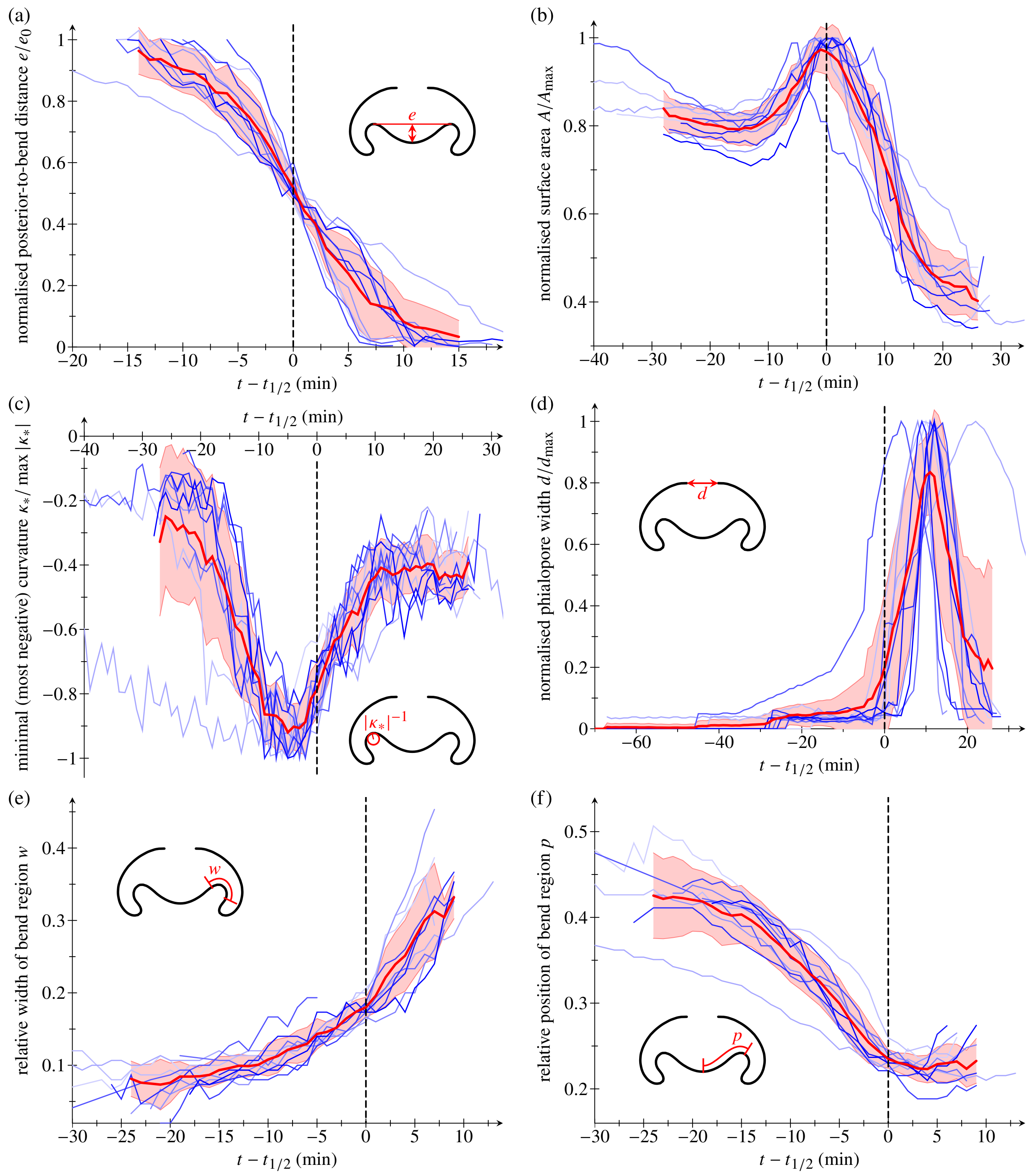

Figure A3. Quantification of 11 Inversion Processes in Real Time. (a) Distance e from the posterior pole to the plane of the circular bend region, normalised by its initial value e_0; (b) Surface area A computed from a surface of revolution obtained from each half of traced outlines, scaled by its maximal value $A_{\max}$; (c) Minimum (most negative) value κ_* of the curvature in the bend region; (d) Diameter of the phialopore d normalised with its maximal value $d_{\max}$; (e) Width w of the bend region (region of negative curvature), normalised by arclength of the traced shape; (f) Position p of the bend region defined as the distance from the posterior pole to the centre of the bend region, normalised by arclength of the traced shape. Measurements (blue lines) on midsagittal embryo outlines are aligned at the time $t_{1/2}$ where e reaches half of its initial value. Averages (red lines) and standard deviations thereof (red shaded areas) are shown for timepoints for which measurements were obtained for at least half of the quantified inversion processes. Insets: cartoons of definitions of e, κ_*, d, w, p. See S1 Data for numerical values.

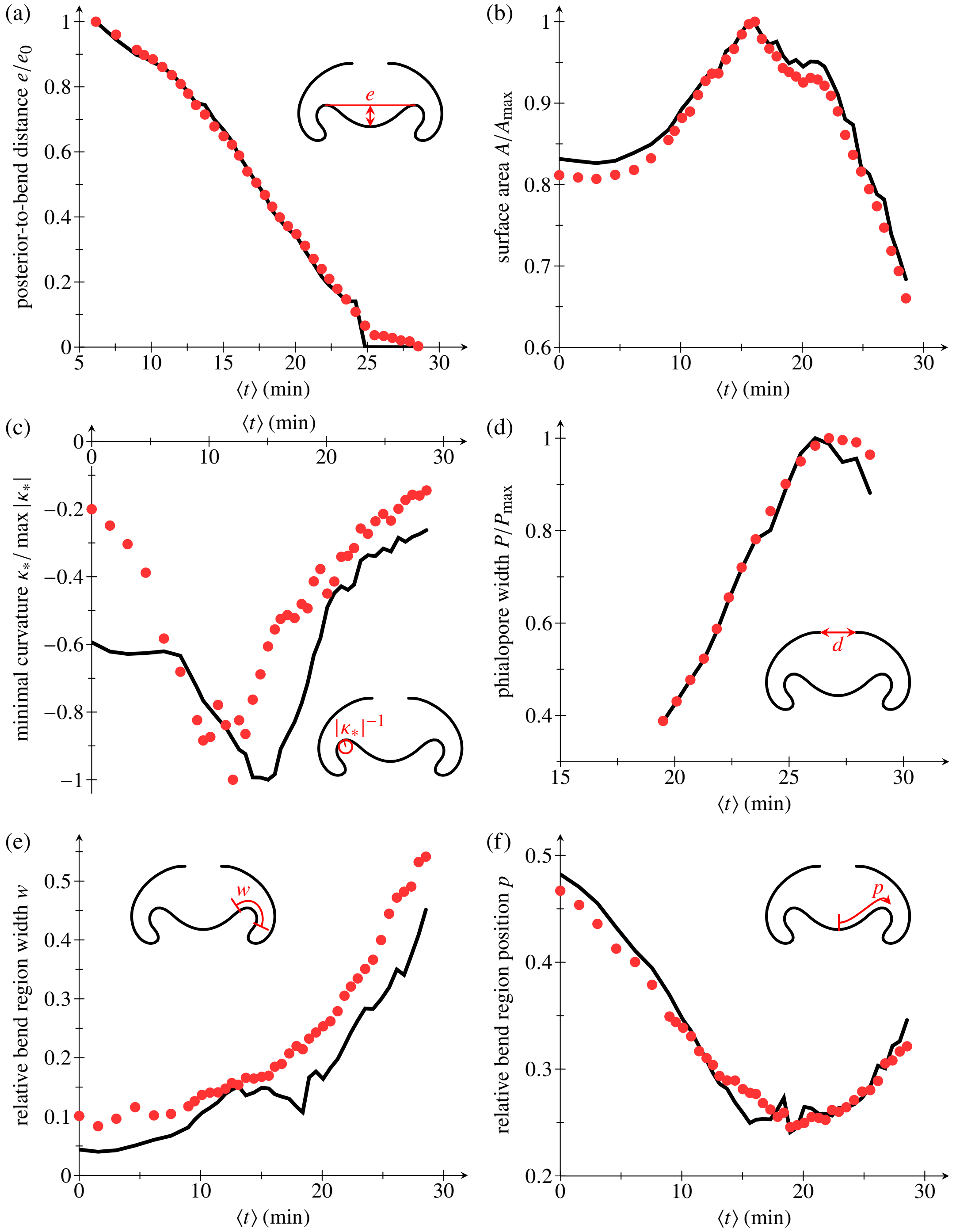

Figure A4. Geometric Descriptors of Fitted Shapes. Geometric descriptors, as in Fig. A3, for the average shapes in Fig. 4 (red marks) and the fits of Fig. 7 (black line). (a) Posterior-to-bend-distance e; (b) Surface area A; (c) Minimum (most negative) value κ_* of the curvature in the bend region; (d) Phialopore diameter d; (e) Bend region width w; (f) Bend region position p. The geometric descriptors are normalised as in Fig. A3; insets provide cartoons of their definitions. See S1 Data for numerical values.

Each of the descriptors evolves in qualitatively similar ways in individual embryos, yet their evolution occurs over different timescales in different embryos and the local maxima in surface area (Fig. A3b) and in phialopore width (Fig. A3d) occurs at different relative times in different embryos. This stresses the variability seen in the summary statistics.

Comparing these geometric descriptors for the experimental averages and the fitted shapes of Fig. 7, we find a good agreement (Fig. A4), although we notice that the fitted shapes underestimate the width of the bend region. Because curvature is a second derivative of shape, it is not surprising that larger differences arise in the minimal bend region

curvature of the average and fitted shapes (Fig. A4).

References

[43] Höhn S, Honerkamp-Smith AR, Haas PA, Khuc Trong P, Goldstein RE. Dynamics of a *Volvox* Embryo Turning Itself Inside Out. Phys Rev Lett. 2015;114:178101. doi:10.1103/PhysRevLett.114.178101.
