# Supplementary material for: The noisy basis of morphogenesis: Mechanisms and mechanics of cell sheet folding inferred from developmental variability
Source: PLoS Biol. 2018 Jul 12;16(7):e2005536. doi: 10.1371/journal.pbio.2005536 (PMC6063725; doi:10.1371/journal.pbio.2005536)
Supplement: S2 Text — Boundary conditions for the contact configuration. Numerical study of the contact configuration. Asymptotic analysis of a toy problem. (PDF) [file pbio.2005536.s012.pdf]

P. A. Haas, S. S. M. H. Höhn, A. R. Honerkamp-Smith, J. B. Kirkegaard, and R. E. Goldstein

S2 Text. Elastic Model in the Contact Configuration.

In this appendix, we analyse the configuration where the rim of the phialopore is in contact with the inverted posterior for completeness of the mechanical analysis. We also analyse a toy problem to illustrate the intricate interplay of geometry and mechanics during contact.

Boundary conditions for the contact configuration. Let P be the angular extent of the axisymmetric phialopore at the anterior pole of the shell. Here, we discuss the contact problem where the shell has deformed in such a way that the rim of the phialopore (at $\theta = \pi - P = Q$, where $\theta = s/R$ is the polar angle) is in contact with the shell at some as yet unknown position $\theta = C$, as shown in Fig. B1a,b.

As in the derivation of the governing equations without contact (Materials and Methods), we shall express the variations in terms of δr and $\delta\beta$. The third variation, δz, is not independent of the former two, and so the condition that the vertical positions of the shell at the point of contact and at the phialopore match must be incorporated via a Lagrange multiplier, U. Ref. [95] raised a related issue in the derivation of the shape equations for vesicles. The Lagrangian for the problem is therefore

$$\mathcal{L} = \mathcal{E} - 2\pi U \int_C^Q f_s \sin\beta \,\mathrm{d}\theta, \tag{B1}$$

where the prefactor has been introduced for mere convenience. We note the variation of Eq. (B1),

$$\frac{\delta\mathcal{L}}{2\pi} = \frac{\delta\mathcal{E}}{2\pi} - \Big[\!\Big[U \tan\beta\,\delta r \Big]\!\Big]_{C_+}^{Q} + U f_s(C_+) \sin\beta(C)\,\delta C + U \int_C^Q \Big\{ f_s \kappa_s \sec^2\beta\,\delta r - f_s \sec\beta\,\delta\beta \Big\}\,\mathrm{d}\theta. \tag{B2}$$

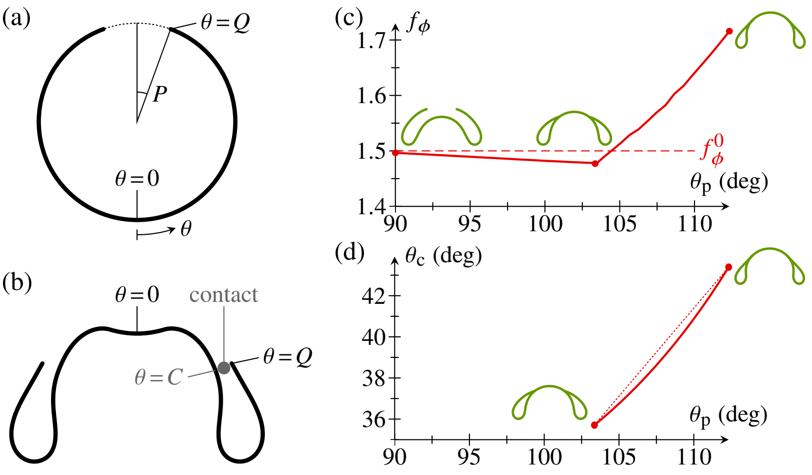

Figure B1. Analysis of the Contact Problem. (a) Undeformed configuration and (b) contact configuration. The phialopore at $\theta = Q = \pi - P$ touches the shell at $\theta = C$, where θ is the polar angle. (c) Increasing circumferential stretch f_ϕ with advancing position θ_p of the peeling front, at constant intrinsic stretch f_ϕ^0. Insets: configuration with inverted posterior (as in Fig. 12d), at beginning of contact, and at a later stage. (d) Advancing contact position with advancing peeling front. Insets: configurations at beginning of contact and at later stage, as in (c).

Next, expanding the condition $\beta(C_-) = \beta(C_+)$ of geometric continuity that we have already implicitly applied in the above, we note that

$$\delta\beta(C_-) + f_s(C_-)\kappa_s(C_-)\,\delta C = \delta\beta(C_+) + f_s(C_+)\kappa_s(C_+)\,\delta C. \tag{B3}$$

Since the outer part of the shell can rotate freely with respect to the inner part at the points of contact, the variations $\delta\beta(C_\pm)$ and $\delta\beta(Q)$ are, by contrast, independent. This is not true of the variations $\delta r(C_\pm)$ and $\delta r(Q)$, however:

$$\delta r(Q) = \delta r(C_-) + f_s(C_-)\cos\beta(C)\,\delta C = \delta r(C_+) + f_s(C_+)\cos\beta(C)\,\delta C. \tag{B4}$$

Analogous expansions were used in Ref. [96] to discuss an adhesion problem for vesicles. Next, a straightforward calculation reveals that the governing equations (24) and (25) remain unchanged if we define $T = -N_s\tan\beta + U\sec\beta/r$ for $C \leqslant \theta \leqslant Q$. For convenience, we adjoin the equation $\mathrm{d}z/\mathrm{d}s = f_s\sin\beta$ to the system (thereby fixing the degree of freedom of vertical translation). The system thus becomes a system of six first-order differential equations on two regions, with two unknown parameters (the contact position C and the Lagrange multiplier U). We thus have to impose fourteen boundary conditions:

$$r(0) = 0, \qquad z(0) = 0, \qquad \beta(0) = 0, \qquad T(0) = 0, \tag{B5a}$$

$$r(Q) = r(C), \qquad z(Q) = z(C), \qquad N_s(Q) = 0, \qquad M_s(Q) = 0, \tag{B5b}$$

as well as the continuity conditions at $\theta = C$,

$$[\![\beta]\!] = 0, \qquad [\![r]\!] = 0, \qquad [\![z]\!] = 0, \qquad [\![M_s]\!] = 0, \tag{B5c}$$

and

$$r(C)[\![N_s]\!]\sec\beta(C) - U\tan\beta(C) = r(Q)N_s(Q)\sec\beta(Q) - U\tan\beta(Q), \tag{B5d}$$

$$[\![T]\!] = -[\![N_s]\!]\tan\beta(C) + \frac{U\sec\beta(C)}{r(C)}, \tag{B5e}$$

$$\frac{[\![\mathcal{E}]\!]}{2\pi} = r(C)[\![f_sN_s]\!] + r(C)M_s(C)[\![f_s\kappa_s]\!]. \tag{B5f}$$

We note that the conditions $r(Q) = r(C)$ and $z(C) = z(Q)$ do not take into account the finite, but small, thickness of the shell. A more detailed condition would require knowledge of the nature of the contact (and is anyway beyond the remit of a thin shell theory).

Numerical study of the contact configuration. We briefly explore shapes in the contact configuration in what follows. We start from a configuration where the posterior hemisphere has inverted, as in Fig. 12d, and advance the peeling front, but now without increasing the intrinsic circumferential stretch f_ϕ^0 at the phialopore. As the peeling front advances, the circumferential stretch at the phialopore increases (Fig. B1c) at constant f_ϕ^0, showing how the phialopore is pushed open by the posterior hemisphere. The procession of the point of contact between the posterior and the phialopore along the inverted posterior speeds up with advancing peeling front position (Fig. B1d) because the closer the point of contact is to the posterior, the more the latter resists the progression of the contact point because of the changing tangent angle.

The inset configurations in Fig. B1c,d also suggest that, as the peeling front advances, the regime of contact at a point discussed here gives way to a second contact regime, where the contact is over a finite extent of the meridian of the shell. We do not pursue this further.

Asymptotic Analysis of a Toy Problem. Some analytic progress can be made and additional insight into the contact configuration can be gained by asymptotic analysis of a toy problem: two elastic spherical shells, an inner shell of radius R_1 and an outer, open shell of radius $R_2 > R_1$, touch at the respective angular positions Θ_1 and $\Theta_2 < \Theta_1$ (Fig. B2a), so that $R_2/R_1 = \sin\Theta_1/\sin\Theta_2$. The intrinsic stretches and curvatures are those of the undeformed shells. For the remainder of this section, we non-dimensionalise distances with respect to the radius R_1 of the inner shell; stresses we non-dimensionalise with Eh.

If the outer shell is moved relative to the inner shell by a distance d (Fig. B2b), the two shells deform in asymptotically small regions near the point of contact. This point of contact moves a distance $d\Xi$ down along the inner shell, determined by matching the displacements of the contact point and the forces exerted by one shell on the other. We assume in particular that the nature of the contact is such that the shells do not exert torques on each other. Since we have non-dimensionalised distances with R_1, our asymptotic small parameter is

$$\varepsilon^2 = \frac{1}{12(1-\nu^2)}\frac{h^2}{R_1^2} \ll 1. \tag{B6}$$

The classical leading-order scalings for this problem are discussed in Ref. [56], for example: deformations are localised to asymptotic inner regions of width $\delta \sim \varepsilon^{1/2}$, in which deviations of the tangent angle from its equilibrium value are of order d/δ, and we assume that $d \ll \delta$. We introduce an inner coordinate ξ, and write the polar angles as $\theta_1 = \Theta_1 + \delta\xi + \mathcal{O}(d)$, $\theta_2 = \Theta_2 + \delta\xi$. We thus expand

$$\beta_1(\theta_1) = \Theta_1 + (d/\delta)b_1(\xi), \qquad \beta_2(\theta_2) = \Theta_2 + (d/\delta)b_2(\xi). \tag{B7}$$

Assuming that $\delta^2 \ll d \ll \delta$, we then have the leading-order expansions

$$N_s^{(1)} = Eh\,\delta d\,\sigma_1(\xi), \qquad N_s^{(2)} = Eh\,\delta d\,\sigma_2(\xi), \tag{B8a}$$

$$N_\phi^{(1)} \overset{(*)}{=} Eh\,E_\phi^{(1)} + \nu N_s^{(1)} = Eh\delta\,a_1(\xi), \qquad N_\phi^{(2)} \overset{(*)}{=} Eh\,E_\phi^{(2)} + \nu N_s^{(2)} = Eh\delta\,a_2(\xi), \tag{B8b}$$

where a_1, a_2 are hoop strains. We note that the relations marked $(*)$ are only valid at leading order, where we may approximate $f_s \approx f_\phi \approx 1$. Let F_r and F_z denote the (suitably scaled) radial and vertical forces exerted by the outer shell on the inner shell. We obtain the leading-order force balances from the energy variation (28):

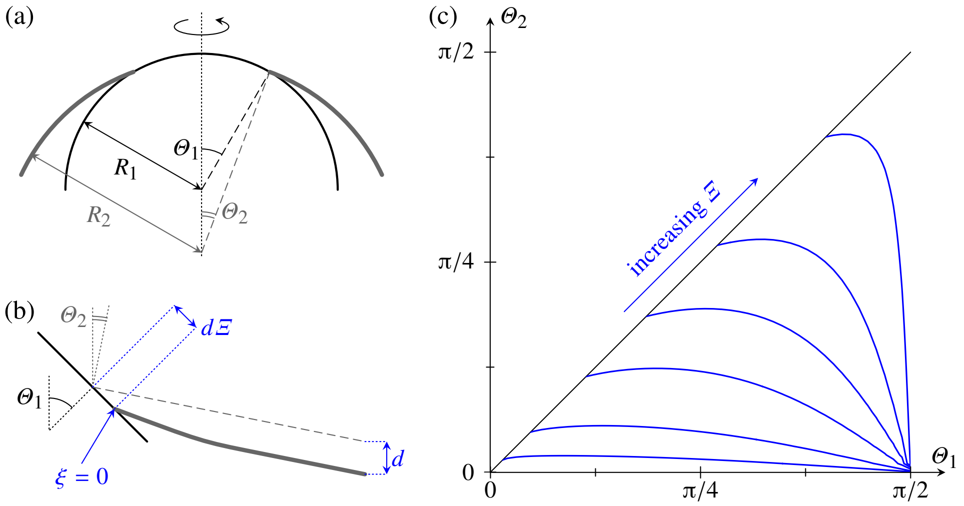

Figure B2. Asymptotic Toy Contact Problem. (a) Two shells of radii R_1 and R_2 are in contact at angular positions Θ_1 and Θ_2, respectively. (b) Relative motion of one shell with respect to the other by a distance d induces deformations of the shell in an asymptotic inner layer of size δ, and causes the point of contact to move by a distance $d\Xi$ along the inner shell. (c) Contours of Ξ in the (Θ_1, Θ_2) plane.

using dashes to denote differentiation with respect to ξ,

$$\sigma_1' \sin^2 \Theta_1 - b_1''' \cos \Theta_1 \sin \Theta_1 = F_z \delta(\xi), \tag{B9a}$$

$$\sigma_1' \sin \Theta_1 \cos \Theta_1 - a_1 + b_1''' \sin^2 \Theta_1 = F_r \delta(\xi). \tag{B9b}$$

This system is closed, at leading order, by the geometric relation $a_1' = -b_1$, as in Ref. [56]. Eliminating σ_1, we obtain

$$b_1'''' + b_1 = (F_r - F_z \cot \Theta_1)\delta'(\xi). \tag{B10}$$

The matching conditions $b_1 \to 0$ as $\xi \to \pm\infty$ reduce the number of undetermined constants to four, which are determined by the jump conditions at the contact point $\xi = 0$.

The asymptotic balance for the outer shell is of course the same, but we must remember that the system has been non-dimensionalised with the radius of the inner shell, for which reason a geometric factor arises in the equations. Thus

$$b_2'''' + \left(\frac{\sin \Theta_1}{\sin \Theta_2}\right)^4 b_2 = 0, \tag{B11}$$

with the matching condition $b_2 \to 0$ as $\xi \to \infty$, leaving two boundary conditions to be imposed on this equation. Since the shells do not exert any moments on each other, $b_2'(0) = 0$. The second condition is obtained from the force balance: the vertical force balance can be integrated once to yield

$$\sin \Theta_1 \sin \Theta_2 \left\{ \sigma_2 - \cot \Theta_2 \left(\frac{\sin \Theta_2}{\sin \Theta_1}\right)^4 b_2'' \right\} = F_z. \tag{B12}$$

Matching to the undeformed, unstressed shell as $\xi \to \infty$ implies $F_z = 0$. The radial force boundary condition resulting from (28) is

$$\sin \Theta_1 \cos \Theta_2 \left\{ \sigma_2(0) + \tan \Theta_2 \left(\frac{\sin \Theta_2}{\sin \Theta_1}\right)^4 b_2''(0) \right\} = F_r, \tag{B13}$$

which, upon imposing (B12), reduces to

$$b_2''(0) = \left(\frac{\sin \Theta_1}{\sin \Theta_2}\right)^3 F_r. \tag{B14}$$

Let $U_r^{(1)}, U_z^{(1)}$ and $U_r^{(2)}, U_z^{(2)}$ denote the respective (non-dimensional) displacements of the contact point $\xi = 0$, scaled with d. Then

$$U_r^{(1)} = \sin \Theta_1 \int_0^\infty b_1 \, d\xi = -\frac{F_r}{2\sqrt{2}} \sin \Theta_1, \tag{B15a}$$

$$U_z^{(1)} = -\cos \Theta_1 \int_0^\infty b_1 \, d\xi = \frac{F_r}{2\sqrt{2}} \cos \Theta_1, \tag{B15b}$$

$$U_r^{(2)} = \sin \Theta_1 \int_0^\infty b_2 \, d\xi = -\sqrt{2} F_r \sin \Theta_1, \tag{B15c}$$

$$U_z^{(2)} = -\sin \Theta_1 \cot \Theta_2 \int_0^\infty b_2 \, d\xi = \sqrt{2} F_r \sin \Theta_1 \cot \Theta_2. \tag{B15d}$$

In particular, these expressions once again contain additional geometric factors resulting from the non-dimensionalisation. The values of the two remaining undetermined constants, F_r and Ξ, are finally obtained by imposing continuity of the displacement of the contact point, i.e.

$$U_r^{(1)} + \Xi \cos \Theta_1 = U_r^{(2)}, \qquad U_z^{(1)} + \Xi \sin \Theta_1 = U_z^{(2)} + 1. \tag{B16}$$

Notice that arclength is computed here from the anterior pole of the shell to match the asymptotic setup of

Ref. [56], and so the 'vertical' axis is pointing downwards in Fig. B2a, giving rise to some sign changes. In particular, we obtain

$$\varXi = \frac{3\sin\varTheta_1}{1 + 2\operatorname{cosec}\varTheta_2 \sin\left(2\varTheta_1 - \varTheta_2\right)}. \tag{B17}$$

The contours of this expression are plotted in Fig. B2c. The very non-linear nature of this expression illustrates that the contact geometry is quite intricate; in particular, $\varTheta_2(\varTheta_1)$ at fixed $\varXi$ is not a monotonic function, but, as expected (since it is easier for the the contact point to slide along the inner shell the more parallel it is to the axis of symmetry), at fixed $\varTheta_1$, $\varXi$ increases with $\varTheta_2$.

References

[56] Haas PA, Goldstein RE. Elasticity and Glocality: Initiation of Embryonic Inversion in *Volvox*. J R Soc Interface. 2015;12:20150671. doi:10.1098/rsif.2015.0671.

[95] Jülicher F, Seifert U. Shape equations for axisymmetric vesicles: A clarification. Phys Rev E. 1994;49(5):4728–4731. doi:10.1103/PhysRevE.49.4728.

[96] Seifert U. Adhesion of vesicles in two dimensions. Phys Rev A. 1991;43(12):6803–6814. doi:10.1103/PhysRevA.43.6803.
